# Effect of endophytic diazotroph *Enterobacter roggenkampii* ED5 on nitrogen-metabolism-related microecology in the sugarcane rhizosphere at different nitrogen levels

**DOI:** 10.3389/fmicb.2023.1132016

**Published:** 2023-08-15

**Authors:** Dao-Jun Guo, Dong-Ping Li, Bin Yang, Krishan K. Verma, Rajesh Kumar Singh, Pratiksha Singh, Qaisar Khan, Anjney Sharma, Ying Qin, Bao-Qing Zhang, Xiu-Peng Song, Yang-Rui Li

**Affiliations:** ^1^College of Life Sciences and Engineering, Hexi University, Zhangye, Gansu, China; ^2^Key Laboratory of Sugarcane Biotechnology and Genetic Improvement (Guangxi), Ministry of Agriculture, Guangxi Key Laboratory of Sugarcane Genetic Improvement, Sugarcane Research Institute, Guangxi Academy of Agricultural Sciences, Nanning, Guangxi, China; ^3^Microbiology Institute, Guangxi Academy of Agricultural Sciences, Nanning, Guangxi, China; ^4^College of Agriculture, Guangxi University, Nanning, Guangxi, China

**Keywords:** *Enterobacter roggenkampii* ED5, sugarcane growth, nitrogen fixation, rhizosphere, metagenomics, nitrogen levels

## Abstract

Sugarcane is an important sugar and energy crop worldwide, requiring a large amount of nitrogen (N). However, excessive application of synthetic N fertilizer causes environmental pollution in farmland. Endophytic nitrogen-fixing bacteria (ENFB) provide N nutrition for plants through biological N fixation, thus reducing the need for chemical fertilizers. The present study investigated the effect of the N-fixing endophytic strain *Enterobacter roggenkampii* ED5 on phytohormone indole-3-acetic acid (IAA), N-metabolism enzyme activities, microbial community compositions, and N cycle genes in sugarcane rhizosphere soil at different N levels. Three levels of ^15^N-urea, such as low N (0 kg/ha), medium N (150 kg/ha), and high N (300 kg/ha), were applied. The results showed that, after inoculating strain ED5, the IAA content in sugarcane leaves was significantly increased by 68.82% under low N condition at the seedling stage (60 days). The nitrate reductase (NR) activity showed a downward trend. However, the glutamine synthase (GS) and NADH-glutamate dehydrogenase (NADH-GDH) activities were significantly enhanced compared to the control under the high N condition, and the *GS* and *NR* genes had the highest expression at 180 and 120 days, respectively, at the low N level. The total N content in the roots, stems, and leaves of sugarcane was higher than the control. The ^15^N atom % excess of sugarcane decreased significantly under medium N condition, indicating that the medium N level was conducive to N fixation in strain ED5. Metagenome analysis of sugarcane rhizosphere soil exhibited that the abundance of N-metabolizing microbial richness was increased under low and high N conditions after inoculation of strain ED5 at the genus level, while it was increased at the phylum level only under the low N condition. The LefSe (LDA > 2, *p* < 0.05) found that the N-metabolism-related differential microorganisms under the high N condition were higher than those under medium and low N conditions. It was also shown that the abundance of *nifDHK* genes was significantly increased after inoculation of ED5 at the medium N level, and other N cycle genes had high abundance at the high N level after inoculation of strain ED5. The results of this study provided a scientific reference for N fertilization in actual sugarcane production.

## Introduction

Sugarcane (*Saccharum* spp.) is the leading sugar and energy crop in China, and its growth requires a large amount of nitrogen (N) (Li and Yang, 2015). N is a nutrient element of many important organic compounds in plants and significantly impacts plant life activities, crop yield, quality, and nutrient accumulation (Novoa and Loomis, 1981; Kraiser et al., 2011). Nowadays, the N nutrition of crops mainly comes from chemical N fertilizers. However, excessive N application also malnourishes soil at a global scale and causes leaching and eutrophication (Zhu and Chen, 2002; Savci, 2012; Qiao et al., 2018). Biological N fixation (BNF) by N-fixing bacteria converts atmospheric nitrogen into ammonia ions available to plants with the action of nitrogenase. The amount of N from BNF in sugarcane reached 40 kg N ha^−1^ annually in Brazil (Urquiaga et al., 2012). Therefore, utilizing BNF can ensure the ecologically sustainable development of sugarcane production. Some endophytic N-fixing strains have already been screened from sugarcane tissues, such as *Beijerinckia, Novosphingobium sediminicola, Pantoea* sp., *Klebsiella, Enterobacter sacchari* sp., *Klebsiella variicola, Gluconacetobacter diazotrophicus*, and *Herbaspirillum* sp. (Dobereiner, 1961; Loiret et al., 2004; Njoloma et al., 2006; Bertalan et al., 2009; Lin et al., 2012b; Zhu et al., 2013; Wei et al., 2014; Muangthong et al., 2015).

The ^15^N isotope dilution technique is commonly used to detect the effects of associated N-fixing bacteria and plants. It was reported that the inoculation of the N-fixing bacteria SC20 contributed 28% of the total N in sugarcane plants determined by the ^15^N isotope dilution technique (Sajjad Mirza et al., 2001). The strains *Bacillus megaterium* CY5 and *Bacillus mycoides* CA1 were found to have an apparent N fixation role in sugarcane by using the ^15^N isotope dilution technique (Singh et al., 2020b). The effect of N-fixing strains including *Microbacterium* sp. 16SH, *Pseudomonas koreensi* CY4, *Kosakonia radicincitans* BA1, and *Stenotrophomonas maltophilia* COA2 on sugarcane has been investigated in China using the ^15^N isotope dilution technique (Lin et al., 2012a; Li et al., 2017; Singh et al., 2020a).

For metagenomics technology, the DNA of microorganism samples taken from the environment is directly extracted to construct a metagenomic library. Genomics research methods are applied to study the genetic composition and community functions of microorganisms contained in environmental samples to reveal the micro-ecological mechanisms in the particular environment at the three levels of community, gene, and function (Hugenholtz and Tyson, 2008; Suenaga, 2012; Escobar-Zepeda et al., 2015; Awasthi et al., 2020). Using metagenomics technology, Pang et al. (2021) found that continuous cropping of sugarcane could change the composition of soil bacteria and fungi, resulting in a reduction of N, total sulfur, cane yield, and sugar content.

Application of an appropriate amount of N fertilizer can effectively regulate the structure and number of soil microbiota, which could support the stability and sustainability of the rhizosphere ecosystem (Dixon and Kahn, 2004; Geisseler and Scow, 2014; Zhao et al., 2019; Hu et al., 2022). In a study, four levels of N with urea, such as zero N (0 kg ha^−1^), low N (96 kg ha^−1^), medium N (482 kg ha^−1^), and high N (964 kg ha^−1^), were applied, but only the low N application significantly increased the richness and diversity of soil bacteria in sugarcane fields (Yang et al., 2021). In another study, medium N application (150 N kg ha^−1^) improved the soil bacterial community in general, and Acinetobacter and Proteobacteria phyla, in particular, were enhanced by 47 and 71%, respectively, compared to the control (Khan et al., 2022).

Our research team isolated the N-fixing strain *Enterobacter roggenkampii* ED5 (strain ED5) from sugarcane roots (Guo et al., 2020). It was found that, after inoculation with the endophytic diazotroph strain ED5, the physiological enzymes and plant hormone levels in sugarcane were significantly enhanced, and some growth-related key genes were enriched at the transcriptional level (Guo et al., 2021, 2022). The rational application of N fertilizer is important in sugarcane production. Using ^15^N isotope dilution technology, we preliminarily proved that strain ED5 has a strong N fixation ability associated with sugarcane. However, it is unknown whether different N levels affect the growth-promoting and N-fixing capabilities of strain ED5 and whether the intervention of ED5 inoculation under different levels of N affects the microecology of the sugarcane rhizosphere.

In this study, ^15^N-urea was used to set up three different N application levels, including low, medium, and high N. Strain ED5 was inoculated in sugarcane at three N levels. The study included (i) analyzing the indole-3-acetic acid (IAA) content in sugarcane, (ii) detecting the N-metabolism-related enzyme activities and gene expressions in sugarcane leaves, (iii) checking the biological N fixation characteristics of sugarcane, and (iv) studying the N-metabolism-related microbial community structure and functional composition and the genes in the N cycle process in sugarcane rhizosphere soil. This aim was to provide a reference for the application of N-fixing bacteria in sugarcane production.

## Materials and methods

The endophytic diazotroph strain ED5 isolated and screened from sugarcane roots (Guo et al., 2020) was used in this study. The low N fixing sugarcane variety GT11 (more N fertilizer is needed for growth than other varieties) was selected. The pot size for the greenhouse experiment was 32 cm in upper diameter, 25 cm in lower diameter, and 27 cm in height. The N fertilizer ^15^N-urea with an abundance of 10.09% used in the experiment was produced by the Shanghai Research Institute of Chemical Industry Co., Ltd., China. The greenhouse experiment was done at the Sugarcane Research Institute of the Guangxi Academy of Agricultural Sciences (GXAAS) with Latitude 108.09834°, Longitude 22.90991°, and altitude 89 m in Nanning, China. The experiment soil was medium-fertility red soil from sugarcane fields in GXAAS.

### Preparation of inoculum liquid and sugarcane seedling transplanting

Strain ED5 was inoculated in LB broth, incubated at 180 rpm and 32°C for 48 h, and centrifuged at 5,000 rpm for 5 min, and the supernatant was discarded. The bacteria were diluted and mixed with sterilized water so that the final concentration of bacteria reached 10^6^ cfu mL^−1^ in the inoculum suspension. Sugarcane for the experiment was planted using the seedling tray method. The length and width of the seedling tray were 40 cm and 30 cm, respectively, and the culture substrate was sterilized by high-pressure steam sterilization. The seed cane was cut into bud segments of the same length, soaked in 1% carbendazim solution for 0.5 h, and then cultured in the tray. The seedlings were transplanted to the greenhouse when they grew to 2–3 leaves and were managed routinely, and those with the same growth momentum were selected for inoculation and transplantation. The root surface of the selected seedlings was washed with sterile water, soaked in the bacteria suspension for 1 h, and then transplanted to the experimental pots. Those soaked in sterile water were used as controls.

### Greenhouse experiment

In the greenhouse experiment, the pot-cultured plants were used to apply ^15^N-urea fertilizer at once after transplanting. Three N application levels were set at low N (0 g ^15^N-urea pot, equivalent to N 0 kg/ha in the field), medium N (1.52 g^15^N-urea per pot, equivalent to N 150 kg/ha in the field), and high N (3.05 g ^15^N-urea per pot, equivalent to N 300 kg/ha urea in the field). The plants were divided into treatments and control groups. The treatment groups were designed as follows: strain ED5 inoculated at low N (GLE), non-inoculated at low N (GL); strain ED5 inoculated at medium N (GME), non-inoculated at medium N (GM); and strain ED5 inoculated at high N (GHE), non-inoculated at high N (GH). A completely randomized block design was adopted with six pots for each treatment, and a total of 108 pots were used for all the treatments.

### IAA content

The IAA content in leaf +1 (Cheavegatti-Gianotto et al., 2011) was determined by the enzyme-linked immunosorbent assay (ELISA) kit produced by Jiangsu Enzymic Immune Industry Co., Ltd., China. The quantity of leaf +1 was weighted, PBS buffer (pH 7.4) was added, and it was ground evenly with a grinder three times, 60 s each time. The product was centrifuged at 4°C at 3,000 rpm for 20 min, and then the supernatant was collected for testing. For standard samples, 50 μl of standard products were injected into each standard well, and a standard curve was drawn. Samples and blank wells were set in the ELISA plate, and 40 μl of diluent and 10 μl of the sample were added for testing. After 100 μl of enzyme-labeled reagent was added to each sample well, the plate was sealed and incubated at 37°C for 60 min. Then the plate sealing film was removed, the liquid was discarded, the washing solution was added and kept for 30 s before the solution was removed, and the wash was repeated five times. The chromogenic reagent was added to each well and cultured in the dark at 37°C for 15 min, then 50 μl of stop solution was added to terminate the reaction, and the OD value was detected with a microplate reader (Spark) at a wavelength of 450 nm. The proteins were quantitated using the BCA kit (Solarbio, China). All the tests were performed with three biological replicates.

### Activities of N-metabolism-related enzymes

The samples of sugarcane leaf +1 were taken at 60, 120, 180, and 240 days after strain ED5 inoculation. The activities of three N-metabolism-related key enzymes, namely, nitrate reductase (NR), glutamine synthase (GS), and NADH-glutamate dehydrogenase (NADH-GDH), were analyzed by kits produced by Jiangsu Edison Biotechnology Co., Ltd., China. All the tests were performed with three biological replicates.

### Expression of N-metabolism-related genes

The expressions of nitrite reductase (*NR*) and glutamine synthase (*GS*) genes in sugarcane leaf +1 were analyzed by quantitative real-time PCR (qRT-PCR) at 60, 120, 180, and 240 days, respectively, after strain ED5 inoculation. The primers for gene amplification are shown in Supplementary Table S1 (Yang et al., 2019). The total RNA in sugarcane leaves was extracted with the plant whole RNA extraction kit (TSP412, Qingke Biology, China), and its integrity and purity were detected by 1.2% agarose gel electrophoresis (1.8 ≤ OD_260_/OD_280_ ≤ 2.1). The Goldenstar RT6 cDNA Synthesis Kit version 2, a reverse transcription kit, was used for RNA reverse transcription amplification. The cDNA products obtained from reverse transcription were diluted with qPCR templates appropriately. The 2×T5 Fast qPCR Mix (SYBR Green I, Qingke Biology, China) was used as the qPCR enzyme, and the expression was detected with a LightCycler 1480 II real-time fluorescence quantitative PCR instrument. The qRT-PCR reaction components are listed in Supplementary Table S2. The 2^−Δ*ΔCt*^ method was used to calculate the relative gene expression (Livak and Schmittgen, 2001).

### Total N content and ^15^N atom %

After inoculation of strain ED5, the total N content and ^15^N atom % in sugarcane roots, stems, and leaves were tested at 60, 120, 180, and 240 days, respectively, after strain ED5 inoculation. The whole sugarcane plants were taken at each testing stage after transplanting, and the soil and impurities on the surface of the roots, stems, and leaves were removed by washing with tap water. The cleaned samples were treated at 105°C for 30 min and then dried at 65°C. The dried sample was crushed into powder for analysis. According to Sáez-Plaza et al. (2013), the Kjeldahl method was used for the total N content measurement. The ^15^N atom % (N enrichment, ^15^N atom) analysis was completed by the Institute of Genetic Physiology, Hebei Academy of Agriculture and Forestry Sciences, China. The ^15^N atom was calculated according to the method suggested by Oliveira et al. (2006). All the tests were performed with three biological replicates.

### Sugarcane rhizosphere soil DNA extraction

Sugarcane rhizosphere soil sample DNA was extracted using the kit method (FastDNA^®^ Spin Kit, MP Biomedicals, United States) at 120 days after inoculating strain ED5. The DNA integrity was checked by 1% agarose gel electrophoresis, and its purity and concentration were analyzed using a NanoDrop2000 (Thermo Fisher Scientific) and TBS-380 (TurnerBioSystems).

### PE library construction and metagenomic sequencing

The NEXTFLEX Rapid DNA Seq Kit (Bio Scientific) was used to construct the PE library. The library molecules were amplified with bridge PCR to generate DNA clusters, and the Illumina NovaSeq second-generation sequencing platform was used for macrogenome sequencing. The raw metagenomic sequencing data have been submitted to the NCBI SRA database with the accession number PRJNA911313.

### Sequence quality control and genome assembly

The fastp software (version 0.20.0) was used to cut the paired-end adapter sequence of the original data, remove low-quality reads, and obtain high-quality reads (Chen et al., 2018). The results are shown in Supplementary Table S3. The optimized quality control sequences were spliced and assembled into contigs (single assembly) using the MEGAHIT (version 1.1.2) software. The final assembly results were screened for contigs ≥300 bp. The assembly results are listed in Supplementary Table S4.

### Gene prediction and construction of a non-redundant gene catalog

The MetaGene software (http://metagene.cb.k.u-tokyo.ac.jp) was used to predict the open reading frames (ORFs) of the contigs in the spliced results (Noguchi et al., 2006), and the gene prediction results are shown in Supplementary Table S5. The genes were selected and translated into amino acid sequences, and the nucleic acid length was equal to or >100 bp. The predicted gene sequences of all the samples (90%, identity; 90%, coverage) were clustered by CD-HIT software (http://www.bioinformatics.org/cd-hit/, version 4.6.1), and the longest gene of each class was taken as the representative sequence to construct a non-redundant gene catalog (Fu et al., 2012). The gene number and length after removing redundancy are listed in Supplementary Table S6.

### Bioinformatics and statistical analysis

Diamond software (http://www.diamondsearch.org/index.php, version 0.8.35) was used to compare the amino acid sequence of the non-redundant gene catalog in the NR, eggNOG (version 4.5.1), COG, and KEGG (version 94.2) databases (BLASTP E-value ≤ 1e-05) (Buchfink et al., 2015). The number of genes belonging to bacteria was used to calculate the bacterial abundance at different taxonomic levels such as domain, kingdom, phylum, class, order, family, genus, and species, and the table of taxonomic abundance levels was constructed accordingly. The gene catalog of N metabolism was constructed according to the KEGG database pathway (ko00910), and the total gene abundance corresponding to KO, EC, and Module was used to calculate the abundance of the corresponding functional categories.

All the data on IAA content, N-metabolism-related enzyme activities, and qRT-PCR were analyzed using Microsoft Excel 2013. The SPSS 22.0 software was used for data processing and statistical analysis, and Duncan's multiple range test was used to analyze variance and multiple comparisons (*p* = 0.05).

## Results

### Total N in sugarcane

**Figure 1:**
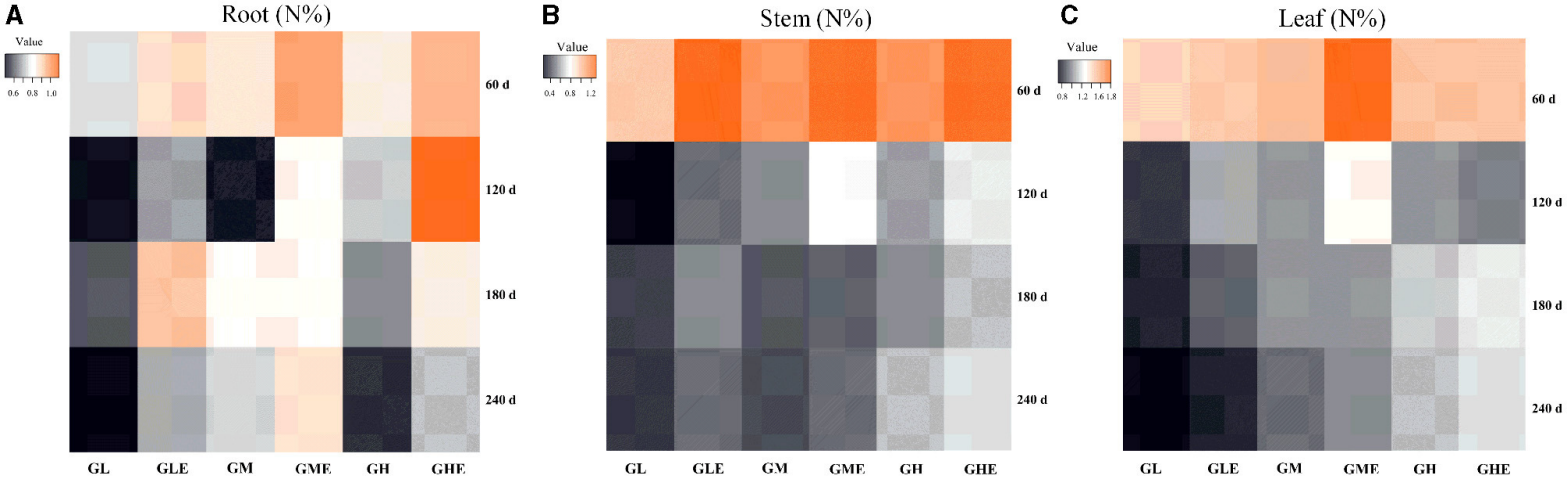
Heatmap analysis of the trend of total nitrogen content changes in sugarcane GT11 inoculated with *E. roggenkampii* ED5. **(A)** Root (N%); **(B)** Stem (N%); **(C)** Leaf (N%). The color intensity in each panel of the heatmap showed the total nitrogen content of sugarcane GT11.

The total N content was significantly increased in sugarcane roots, stems, and leaves inoculated with strain ED5 at all stages compared with the non-inoculated plants (Figure 1; Supplementary Table S7). At the medium N level, it showed no significant difference at 180 days but was significantly enhanced at other stages. However, the total N content in sugarcane tissue was significantly decreased at the high N level, except for the stems and leaves at 180 and 240 days (Supplementary Table S7). Overall, the total N content in sugarcane showed an increasing trend compared to the control.

### ^15^N atom %

The results of the ^15^N atom % in sugarcane are listed in Table 1. The ^15^N atom % in all the organs of sugarcane was significantly decreased compared to the control after strain ED5 inoculation at the medium N level, and the highest loss was observed at 60 days with 24.71% in roots, 26.69% in stems, and 22.01% in leaves, respectively (Table 1). At the high N level, there was no significant difference in ^15^N atom % in roots among different stages. However, it increased by 8.25 and 11.73% in stems and leaves, respectively, at 60 days, and there was no significant change at 240 days (Table 1). The results showed that the low-dose N fertilizer was more conducive to the BNF effects of strain ED5 on sugarcane, and the high dose of N fertilizer may inhibit the function of BNF.

**Table 1:** Effect of *E. roggenkampii* ED5 on ^15^N atom % of sugarcane at different nitrogen application levels.

**Sugarcane organs**	**Time (days)**	**Medium nitrogen**		**High nitrogen**	
		**GM**	**GME**	**GH**	**GHE**
Root (^15^N atom %)	60	3.367 ± 0.234^b^	2.535 ± 0.184^c^	4.591 ± 0.249^a^	4.578 ± 0.210^a^
	120	2.580 ± 0.099^b^	2.136 ± 0.158^c^	3.910 ± 0.015^a^	3.719 ± 0.065^a^
	180	2.311 ± 0.503^b^	1.599 ± 0.072^c^	3.290 ± 0.011^a^	3.559 ± 0.045^a^
	240	1.822 ± 0.040^b^	1.489 ± 0.104^c^	2.941 ± 0.049^a^	2.881 ± 0.040^a^
Stem (^15^N atom %)	60	3.774 ± 0.248^c^	2.88 ± 0.094^d^	4.826 ± 0.088^b^	5.224 ± 0.016^a^
	120	2.709 ± 0.058^b^	2.559 ± 0.064^c^	4.304 ± 0.007^a^	4.277 ± 0.007^a^
	180	2.278 ± 0.020^c^	1.788 ± 0.028^d^	4.166 ± 0.053^a^	3.828 ± 0.076^b^
	240	1.892 ± 0.047^b^	1.656 ± 0.072^c^	2.838 ± 0.041^a^	2.676 ± 0.109^a^
Leaf (^15^N atom %)	60	3.539 ± 0.099^c^	2.760 ± 0.136^d^	4.526 ± 0.081^b^	5.057 ± 0.057^a^
	120	2.657 ± 0.063^b^	2.445 ± 0.105^c^	4.183 ± 0.003^a^	4.182 ± 0.008^a^
	180	2.156 ± 0.062^c^	1.650 ± 0.087^d^	3.878 ± 0.030^a^	3.637 ± 0.082^b^
	240	1.938 ± 0.053^b^	1.717 ± 0.170^c^	3.114 ± 0.023^a^	3.081 ± 0.046^a^

The data are presented as mean ± SE (*n* = 3, Duncan's multiple range test). The means with the same alphabets in the same line are not significantly different at *p* < 0.05.

### IAA content in sugarcane leaves

**Figure 2:**
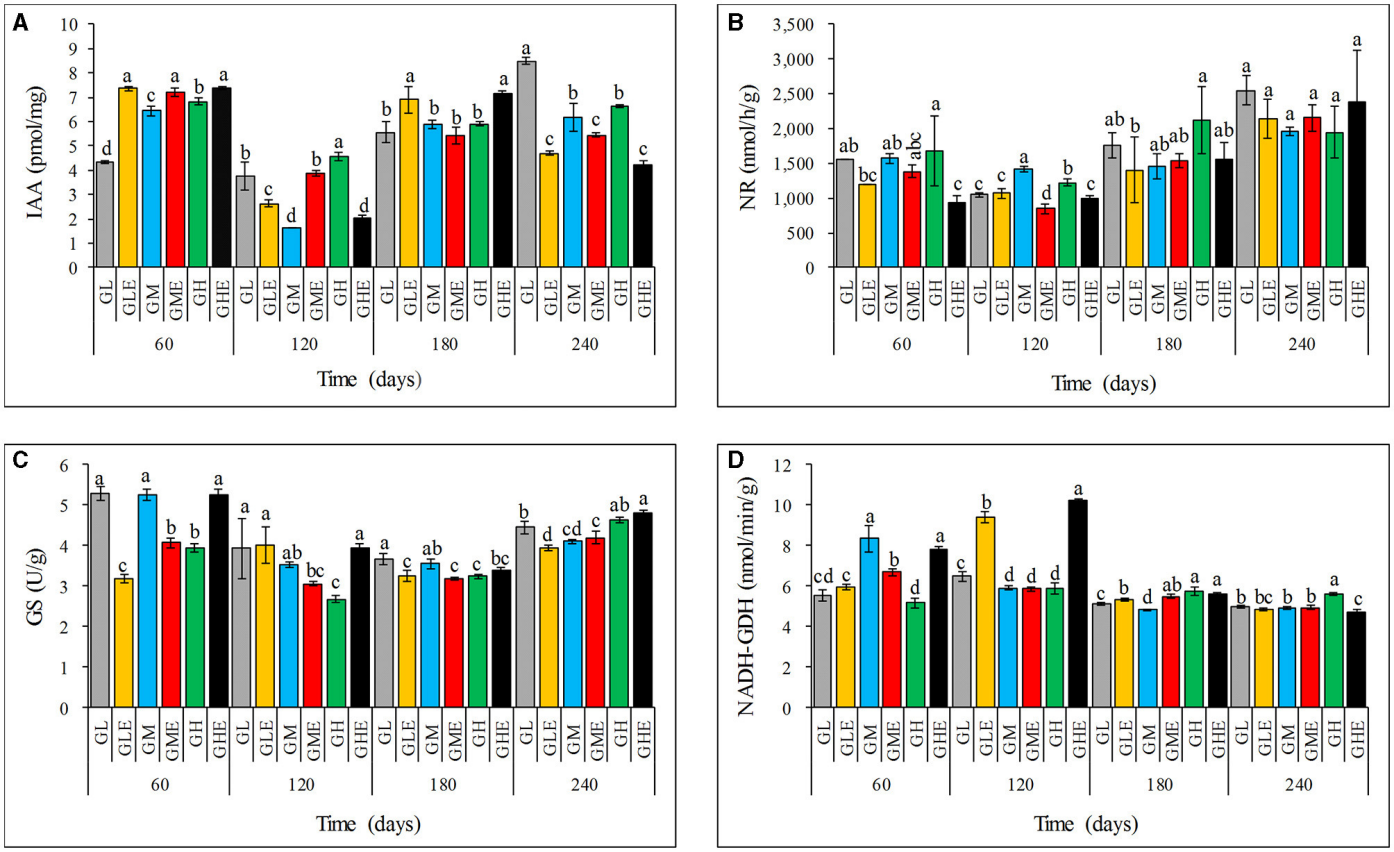
Effect of *E. roggenkampii* ED5 on the IAA contents and activities of N-metabolism-related enzymes in leaves of sugarcane GT11 at different nitrogen application levels. **(A)** IAA; **(B)** NR; **(C)** GS; **(D)** NADH-GDH. The same letter indicates no significant difference was detected at Duncan's multiple range test, *P* ≤ 0.05 (*n* = 3).

The IAA content in sugarcane leaves after inoculation with strain ED5 showed a diverse trend (Figure 2). At the low N level, the IAA content increased by 69.82 and 23.91% at 60 days and 180 days, respectively, but decreased dramatically at 120 and 240 days. At the medium N level, the IAA content increased by 11.78 and 136.59% at 60 and 120 days but decreased at 180 and 240 days. The IAA content at low and medium N application levels has the same trend (Figure 2A). The results of this study indicated that the IAA content in sugarcane at the seedling stage (60 days) increases at different N levels, so it is suggested that the sugarcane seedling stage may be the key stage for strain ED5 to promote plant growth.

### N-metabolism enzyme activities

This study assessed the activities of N-metabolism-related enzymes, NR, GS, and NADH-GDH (Figure 2). It was found that the NR activity showed an overall downward trend under different N levels at all the growth stages (Figure 2B). Similarly, the GS enzyme activity exhibited a downward trend at both low and medium N levels but an upward trend at the high N level compared to the control. At the high N level, the GS activity increased significantly by 42.14% at 60 days (Figure 2C). The NADH-GDH activity was significantly higher than that in the control, except for that at 240 days. The highest increase in NADH-GDH activity was 45.22% at 120 days at the low N level (Figure 2D). The NADH-GDH activity exhibited an opposite trend at medium and high N levels compared to the control, which was decreased at the low N level but enhanced at the high N level at 60 days (Figure 2D). Therefore, the high N condition significantly improved the GS activity in sugarcane after inoculating strain ED5. Therefore, it is important to improve the NADH-GDH activity and decrease the NR activity at low N conditions, especially at the seedling stage (60 days).

### Expression of NR and GS genes

**Figure 3:**
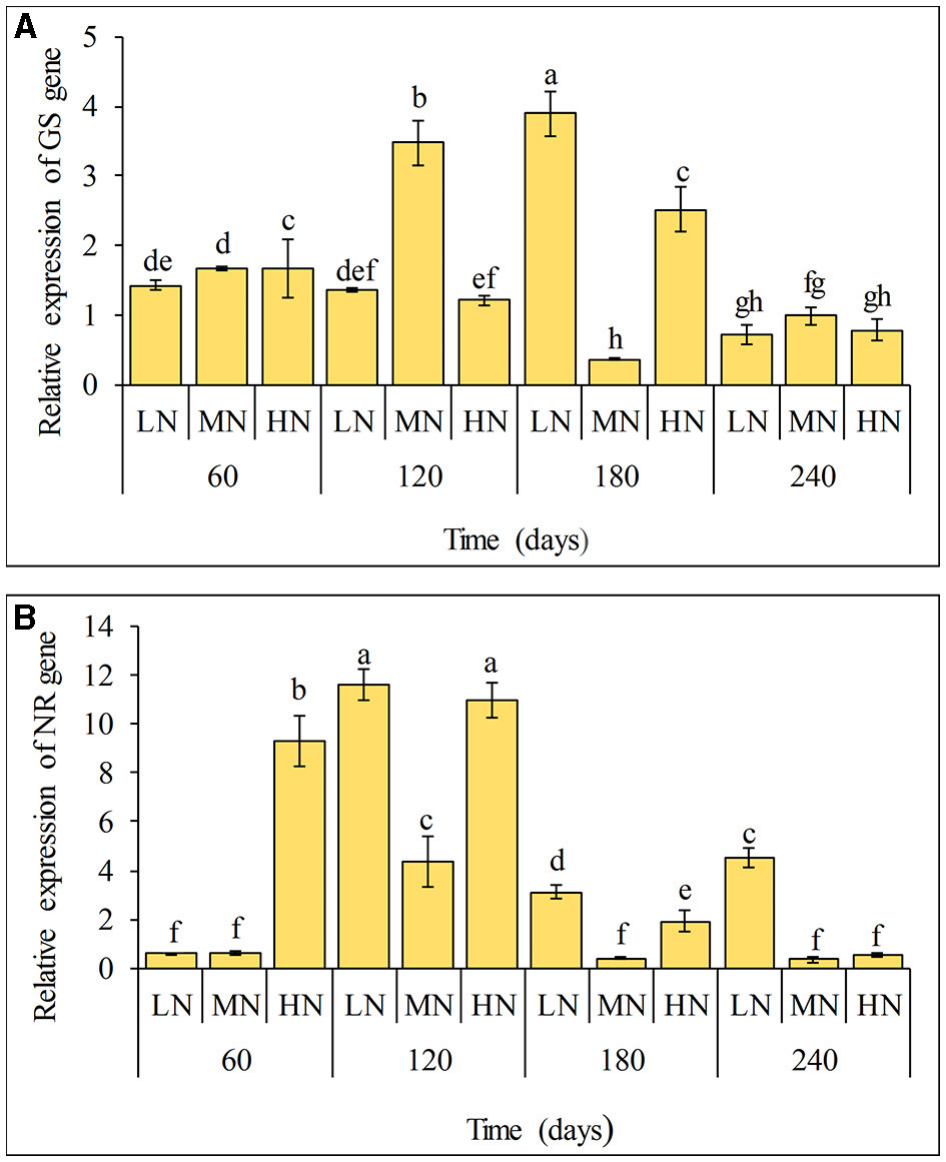
Expression of NR and GS genes in leaves of sugarcane GT11 at different nitrogen application levels. **(A)** GS; **(B)** NR. The same letter indicates no significant difference was detected at Duncan's multiple range test, *P* ≤ 0.05 (*n* = 3).

The expressions of qRT-PCR analysis of GS and NR coding genes in sugarcane after inoculation with strain ED5 at different N levels are presented in Figure 3. It was found that the gene *GS* had the highest expression at 180 days. However, the expression of the gene *NR* was significantly increased at 120 days with the highest expression at the low N level (Figure 3). This showed that the strain ED5 might significantly impact sugarcane N metabolism under low N conditions.

### N-metabolism-related microbial community structure

**Figure 4:**
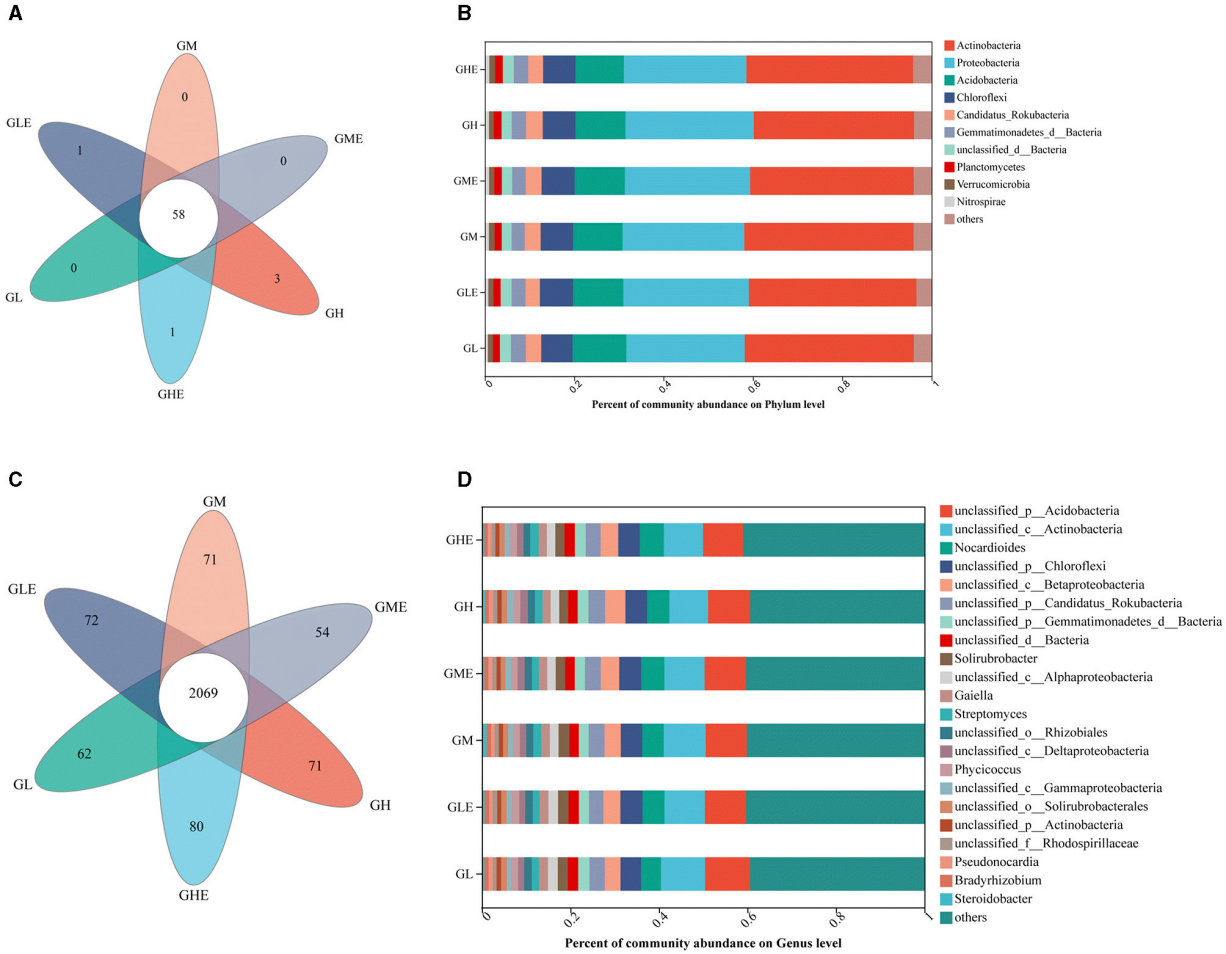
N-metabolism-related microbial community structures in sugarcane variety GT11 rhizosphere soil at different nitrogen application levels. **(A, B)** On the phylum level; **(C, D)**, on the genus level.

The N-metabolism-related gene catalog was constructed according to the KEGG database pathway (ko00910), and the microbial annotation was performed in the NR database, with an abundance of < 0.01 classified as others. The results (Figure 4) showed that, in total, the microorganisms in the GEL and GHE groups were annotated in 59 phyla, including one unique phylum at the phylum level, while 61 phyla containing three unique phyla were annotated in the GH group and 58 phyla with no unique phylum were annotated in the GL, GM, and GME groups (Figure 4A). There were nine phyla in the rhizosphere soil of sugarcane at different N levels, which were *Actinobacteria* (35.89–37.93%), *Proteobacteria* (26.47–28.69%), *Acidobacteria* (10.84–11.26%), *Chloroflexi* (7.07–7.42%), *Rokubacteria* (3.23–3.76%), *Gemmatimonadetes* (2.92–3.33%), *Planctomycetes* (1.58–1.74%), *Verrucomicrobia* (1.10–1.27%), and *Nitrospirae* (0.71–1.02%) (Figure 4B). At the genus level, there were 17, 15, 14, 12, 12, and 21 unique genera in the GL, GLE, GM, GME, GH, and GHE groups, respectively (Figure 4C). It means there was higher microbial richness after inoculation with strain ED5 at a high N level. Additionally, 22 different genera related to N metabolism were detected, of which 14 belonged to unclassified genera, and the other eight genera were *Nocardioides* (4.53–5.25%), *Solirubrobacter* (2.01–2.44%), *Gaiella* (1.82–2.02%), *Streptomyces* (1.79–1.94%), *Phycicoccus* (1.42–1.52%), *Pseudonocardia* (0.78–1.03%), *Bradyrhizobium* (0.71–1.01%), and *Steroidobacter* (0.58–1.09%) (Figure 4D).

### Differences in abundance of N-metabolism-related microorganisms

**Figure 5:**
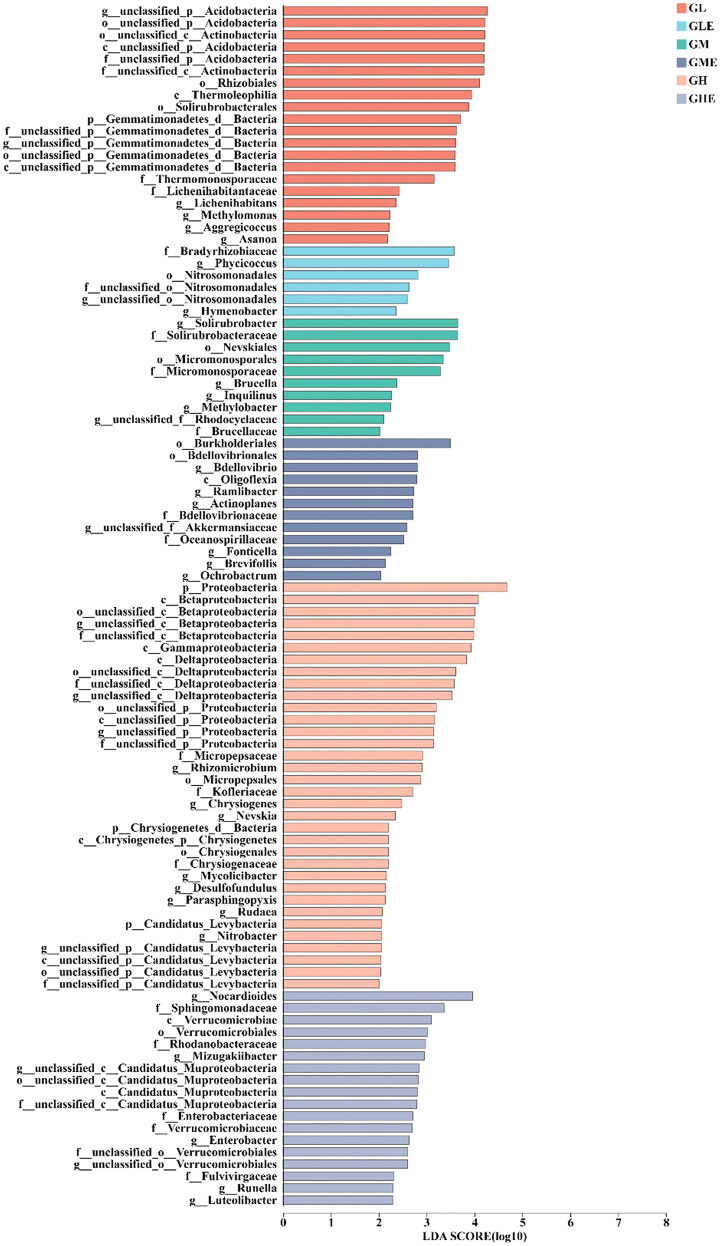
The LEfSe bar chart of N-metabolism-related differences in taxon abundance in the rhizosphere soil of sugarcane GT11 at different nitrogen levels.

This study used the LefSe (LDA >2, *p* < 0.05) bar chart to analyze the N metabolism in various microorganisms from phylum to genus. A total of 135 differentially abundant taxonomic clades were identified, resulting in divergences in different treatments (Figure 5, Supplementary Table S8). The representative differentially abundant microbial taxa included *Proteobacteria* (LDA = 4.57, *p* = 0.048), *Betaproteobacteria* (LDA = 4.04, *p* = 0.038), *Gammaproteobacteria* (LDA = 3.82, *p* = 0.039), and *Deltaproteobacteria* (LDA = 3.66, *p* = 0.037), which were enriched in the GH group. In the GHE group, *Nocardioides* (LDA = 3.82, *p* = 0.028), *Sphingomonadaceae* (LDA = 3.26, *p* = 0.041), *Verrucomicrobiales* (LDA = 3.07, *p* = 0.033), and *Mizugakiibacter* (LDA = 2.93, *p* = 0.017) were found as the representative differentially microbial taxa. Differently abundant microorganisms, including *Acidobacteria* (LDA = 4.12, *p* = 0.041), *Rhizobiales* (LDA = 3.91, *p* = 0.048), *Thermoleophilia* (LDA = 3.80, *p* = 0.016), and *Solirubrobacterales* (LDA = 3.75, *p* = 0.018) were present in the GL group. Differentially abundant microbial taxa of *Bradyrhizobiaceae* (LDA = 3.45, *p* = 0.034) and *Phycicoccus* (LDA = 3.26, *p* = 0.043) were found in the GLE group. *Solirubrobacter* (LDA = 3.60, *p* = 0.030) and *Nevskiales* (LDA = 3.49, *p* = 0.035) were enriched in the GM group, and *Burkholderiales* (LDA = 3.42, *p* = 0.050) and *Bdellovibrionales* (LDA = 2.70, *p* = 0.017) were found in the GME group. These results indicated that there were more N-metabolism-related microbial abundant taxonomic clades in the rhizosphere soil of sugarcane under the high N condition than in medium and low N conditions, and those in the control group were higher than those in the strain ED5 inoculation group.

### N-metabolism-related functional composition

**Figure 6:**
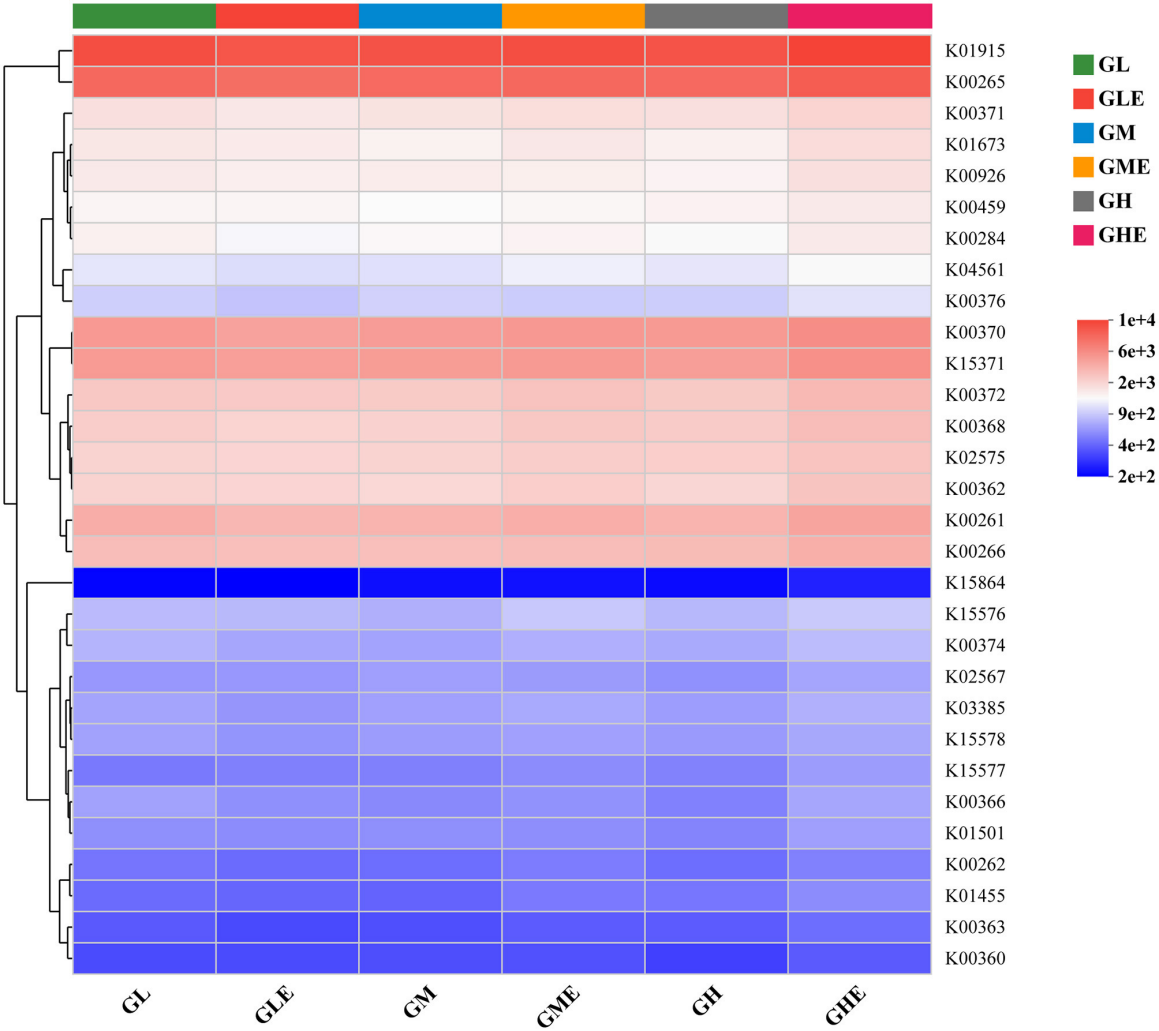
Heatmap analysis showing functions of N-metabolism-related microorganisms in the rhizosphere soil of sugarcane GT11 at different nitrogen application levels. The color intensity in each panel of the heatmap showed the functional abundance of KO.

The top 30 classes of KEGG and KO hierarchical N-metabolism-related functional composition in the rhizosphere soil of sugarcane were analyzed by heatmap. The results (Figure 6) showed that the abundance of glutamine synthetase (K01915), glutamate synthase (GOGAT) large chain (K00265), NR (K00370), and GDH (K15371) encoding genes was higher than that of other genes (Figure 6). However, the KO function of carbonic anhydrase (K01673) had lower gene abundance in the GM and GH groups, and hydroxylamine reductase (K15864) had the lowest gene abundance in all the treatment groups. Overall, the KO functional composition for N metabolism in the rhizosphere soil of sugarcane was similar in different treatment groups.

### Comparison of N-cycle process genes

**Figure 7:**
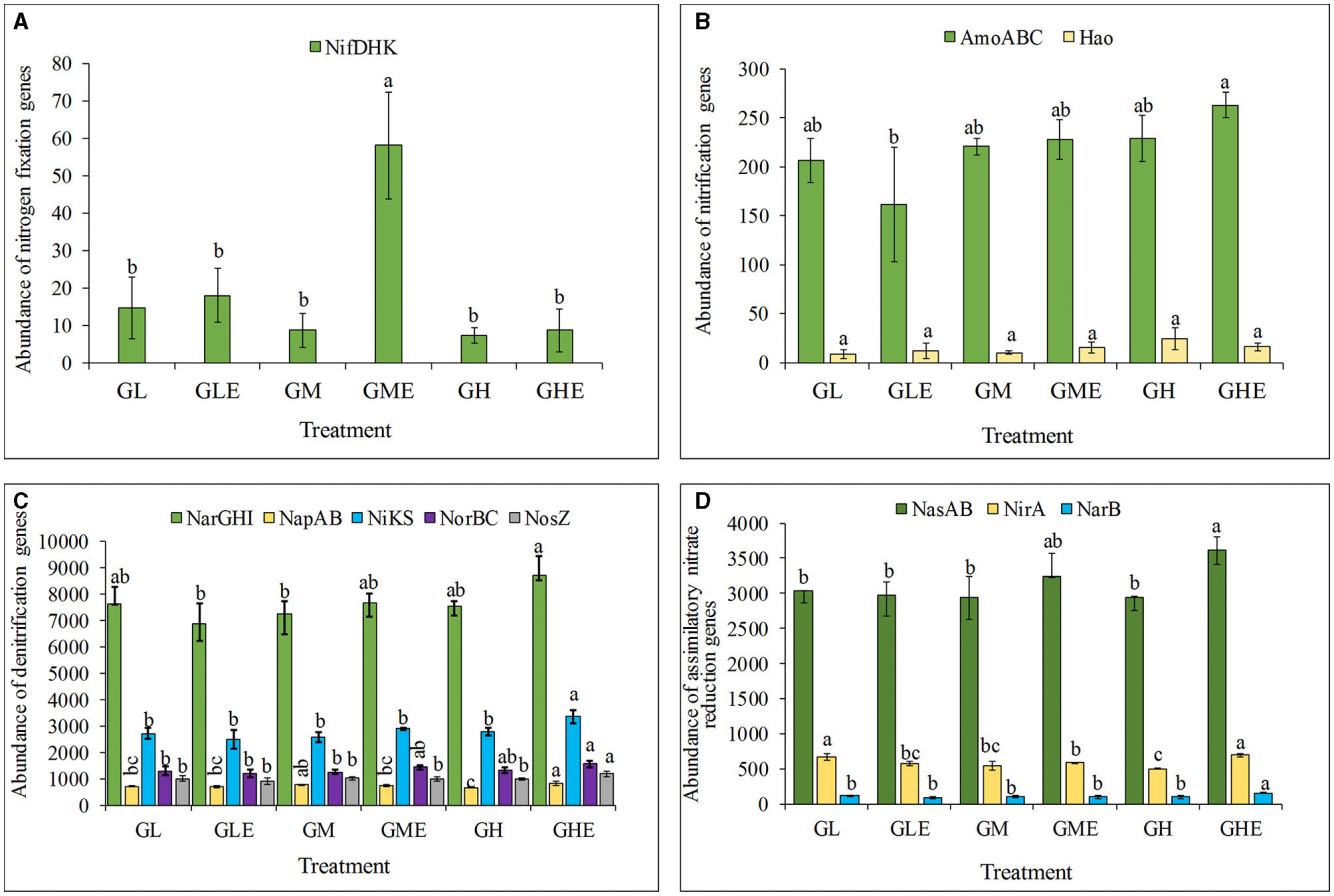
The abundance of nitrogen fixation functional genes in the rhizosphere of sugarcane GT11. **(A)** Nitrogenase genes; **(B)** nitrification process genes; **(C)** denitrification process genes; **(D)** nitrate assimilation and reduction to ammonia-related genes. The same letter indicates no significant difference was detected at Duncan's multiple range test, *P* ≤ 0.05 (*n* = 3).

The abundance of the N-fixing genes *nifDKH* was analyzed in the rhizosphere soil of sugarcane at different N levels. The results (Figure 7) showed that the abundance of *nifDKH* genes in the group inoculated with strain ED5 was significantly higher than the control group by 571.41% at medium N level. However, other treatment groups had no significant difference in N-fixing gene abundance (Figure 7A). The abundance of ammonia monooxygenase-encoding genes *AmoABC* was significantly lower by 21.93% than that in the control group at the low N level. However, it was substantially higher by 15.35%, although it was not statistically significant at the medium N level (Figure 7B). The nitrate reductase gene *NarGHI* in sugarcane rhizosphere soil was significantly enhanced at medium and high N levels by 5.37 and 15.21%, respectively, and significantly reduced by 9.66% compared to the control at the low N level. The abundance of nitrate reductase (cytochrome)-encoding genes *NapAB* decreased considerably by 7.09% at medium N level and increased by 24.67% compared to control at low N level. The abundance of the nitrite reductase (NO^−^)-coding gene *NiKS* was significantly increased by 19.99% at the high N level. In contrast, there was no significant difference in low and medium N conditions compared to the control. Additionally, the abundance of the NO reductase subunit-encoding gene *NorBC* and the N_2_O reductase-encoding gene *NosZ* was significantly increased under the high N condition (Figure 7C). The assimilative nitrate reductase catalytic subunit encoding genes *NasAB* and nitrite ferredoxin reductase-encoding gene *NirA* were increased significantly, with increments of 10.18 and 8.55% at medium level and 22.99 and 38.58% at the high N level. The abundance of the ferredoxin-nitrate reductase-encoding gene *NarB* was significantly increased by 56.75% at the high N level as compared to the control (Figure 7D). Based on these results, the medium N condition might improve the N fixation activity in sugarcane rhizosphere soil microorganisms after strain ED5 inoculation. The high N condition had a significant impact on the processes of nitrification, denitrification, and nitrate assimilation and reduction to ammonia in sugarcane rhizosphere soil after inoculation of strain ED5.

## Discussion

Nitrogen (N) nutrition is critical to improve sugarcane productivity. However, excessive application of chemical N fertilizer pollutes the environment and increases the cost of sugarcane production (Bordonal et al., 2018; Sanches and Otto, 2022). Utilizing the N-fixing function of microorganisms is an ecological way to reduce the amount of chemical N fertilizer (Shridhar, 2012). It was reported that the combined application of N fertilizer and *Bacillus pumilus* TUAT-1 improved rice yield and growth parameters and enhanced the ability of rice to withstand salinity stress (Win et al., 2022). Under different doses of N fertilizer, PGPB strains *Azospirillum brasilense* and *Bacillus circulans* were inoculated with stevia, which improved soil quality and nutrient uptake and significantly increased the content of steviol glycosides (Alotaibi et al., 2022). Combining N fertilizer and azotobacter can improve wheat yields more than azotobacter or N fertilizer alone (El-Sorady et al., 2022).

At present, the biological N fixation in rice, sugarcane, maize, soybean, wheat, and other crops has been studied extensively (Baldani et al., 2002; Ladha and Reddy, 2003; Ciampitti and Salvagiotti, 2018; Rosenblueth et al., 2018; Sheoran et al., 2021). The ^15^N isotope dilution technique is a common research method for biological N fixation in plants (Carranca et al., 1999). Five mungbean genotypes were inoculated with *Bradyrhizobium* spp. under greenhouse conditions, and it was found that different soil properties significantly affected the N fixation effect and plant growth (Diatta et al., 2020). Reinprecht et al. (2020) reported that applying N fertilizer reduced the symbiotic N fixation effect of *Rhizobium leguminosarum* and soybean. Using the ^15^N isotope dilution method, Hardarson et al. (1991) found that the N_2_ fixation rate was slightly reduced to 75% at the 100 kg N ha^−1^ application. In contrast, it will be further reduced to 60 and 43% at 200 and 400 kg N ha^−1^ application conditions, respectively. The N fixation characteristics of *Lupinus angustifolius* cv. Iliyarrie were evaluated on different N application levels of ammonium nitrate (60, 90, 100, 120, and 150 kg N ha^−1^), and it was shown that mineral N replaced biologically fixed N, thereby reducing the soil N use efficiency of *Lupinus angustifolius* cv. Iliyarrie N fixation with an average loss of 33 kg N ha^−1^ (Evans et al., 1987). The biomass and N use efficiency of maize and sorghum were significantly improved by inoculating PGPB under zero N fertilization; however, the dry stem quality and N fixation efficiency were enhanced under N fertilizer application (Aquino et al., 2021). In the present study, the percentage of total N in the roots, stems, and leaves of sugarcane inoculated with strain ED5 was significantly increased, and the ^15^N atom % was considerably lower than the control at medium N level, indicating that strain ED5 has a good function of N fixation in sugarcane.

The secretion of endogenous plant hormones by the PGPB is a direct cause of plant growth-promoting effects. The IAA content in sugarcane was significantly enhanced after inoculation with strains *Streptomyces chartreusis* WZS02, *Pseudomonas aeruginosa* B18, and *Gluconacetobacter diazotrophicus* PAL5, and plant growth was promoted (Ullah et al., 2019; Wang et al., 2019; Singh et al., 2021). The strain PS3 *Rhodopseudomonas palustris* was inoculated into *Brassica rapa* var. *chinensis* at hydroponic conditions with nitrate as the primary N source, which significantly increased the endogenous IAA level in the plant leaves (Hsu et al., 2021). Our previous studies found that strain ED5 can efficiently secrete IAA *in vitro* and reach 732.93 μg mL^−1^ under 5% tryptophan condition (Guo et al., 2020). In this study, the N participation significantly affected the changes in IAA content in sugarcane compared to the control, indicating that strain ED5 may regulate sugarcane growth by secreting IAA.

NR, GS, and NADH-GDH enzymes are vital for N metabolism in plants. In the present study, the activities of N-metabolism-related enzymes NR, NADH-GDH, and GS in sugarcane were significantly affected by inoculating strain ED5, among which the NR activity was increased considerably under medium N condition compared to the control at the late growth stage of sugarcane. In contrast, the GS activity was significantly higher than that in the control at different stages. dos Santos et al. (2020) found that the N-assimilating enzymes NR and GS activities were affected by inoculation with five kinds of N-fixing bacteria. The activity of GS was higher than that in the control. In contrast, the NR activity showed variations. The activities of enzymes related to cucumber N metabolism (NR, GS, and GDH) were increased after PGPB strain *Bacillus tequilensis* SX31 inoculation (Wang et al., 2022). Similar to this study, diazotrophic *Paenibacillus beijingensis* BJ-18 was inoculated in maize, cucumber, and wheat with different N fertilization levels, and the results showed that N uptake, nitrate transporter (NRT), and NiR (sub-NR), NR, GS, and GOGAT were upregulated by 1.5–91.9-fold (Li et al., 2019). The relative expression of NR and GS genes in sugarcane leaves analyzed using qRT-PCR showed that the effects of strain ED5 on gene expression were different at different N levels.

The soil N-metabolism cycle plays a vital role in the plant ecosystem, in which a large number of microorganisms and plants interact to catalyze the redox reaction of N compounds, and microbes are the main drivers for maintaining the N cycle in the rhizosphere of plants (Knops et al., 2002; Chapman et al., 2006; Isobe and Ohte, 2014; Coskun et al., 2017; Moreau et al., 2019; Ramm et al., 2022). The application of N fertilizer can change the microbial community in the plant rhizosphere, directly affecting the N cycle (Nemergut et al., 2008; Dincá et al., 2022). Some studies reported that microbial fertilizer preparations could effectively change the structure of soil microbial communities, improve the ecological environment of plant rhizosphere microorganisms, and indirectly promote plant growth (Chen et al., 2011; Elnahal et al., 2022). N addition was the main driving force of soil microecological change. The microbial diversity will be reduced by long-term N addition. The soil acidification caused by N application is the crucial reason for the decline of soil microbial diversity because bacteria are suitable for surviving in a neutral environment, and N addition reduces bacterial diversity (Dai et al., 2018; Yang et al., 2020). In this study, the microbial richness of N-metabolizing bacteria in the sugarcane rhizosphere was improved under the high N fertilization condition, indicating that the high N condition is more conducive to the survival of N-metabolism-related bacteria. In an experiment, tomatoes were inoculated with *Bacillus pumilus* (PGPB) and planted with native soil without N fertilization and 150 mg N kg^−1^ urea soil in pots, which increased plant growth, N uptake, soil ${\text{NH}}_{4}^{+}$ concentration, rhizosphere bacterial population, soil bacterial gene expression, and soil nitrogenase activity (Masood et al., 2020). According to previous reports, the contents of physiological and chemical factors such as available phosphorus, N, and pH in soil are related to the abundance of N-fixing bacteria (Orr et al., 2012; Che et al., 2018). The associative N-fixation process of N-fixing bacteria and plants requires more energy, and organic carbon is the main energy source for N-fixing bacteria in soil (Herbert, 1999). However, applying N fertilizer can increase the organic carbon content, so the abundance of N-fixing genes and the diversity of N-fixing bacteria in the soil could be increased (Rodrigues Coelho et al., 2008). N fertilizer addition increased the number and diversity of endophytic diazotrophic bacterial communities in maize, while there was no significant difference in the rhizosphere soil (Rodríguez-Blanco et al., 2015). After diazotrophic *Paenibacillus triticisoli* BJ-18 was inoculated in maize on different N fertilization levels, the abundance and diversity of the bacterial community and diazotrophic community were significantly higher in the rhizosphere than in roots and stems through 16S *rRNA* and *nifH* gene sequencing (Li et al., 2021b).

Excessive application of N fertilizer can reduce the abundance of N-fixing genes and microbial richness in crops (Wu, 2011) and the abundance of the nitrogenase gene *nifH* in upland sorghum (Rodrigues Coelho et al., 2008). It was also found that N fertilization did not affect the abundance of the nitrogenase *nifH* gene in the farmland soil (Ouyang et al., 2018). The effects of diazotrophic microbial taxa on the abundance, activity, diversity, and community composition of grass taxa (switchgrass and big bluestem) rhizosphere soil microbes were evaluated under different N application conditions (0, 67, and 202 kg N ha ^−1^), and the results showed that the diazotrophic microbial taxa were more vital in grass green-up, initial harvest, and second harvest agricultural seasons. The abundance of *nifH* transcripts of diazotrophic microbial taxa was reduced at excessive fertilization (202 kg N ha^−1^) compared to moderate fertilization (67 kg N ha^−1^), which reflected that high N fertilization has an inhibitory effect on soil diazotrophic microbial richness (Hu et al., 2021). The diversity of diazobacteria in red soil with long-term N fertilization was analyzed by PCR-RFLP, and the results showed that the diversity of *nirK* and *nosZ* genes was lower than that of *nifH*, but the sensitivity of the *nirK* and *nosZ* genes was higher than that of the *nifH* gene under different N fertilization conditions (Zhang et al., 2021). Similarly, long-term N addition significantly reduced the abundance of *nifH* and 16S *rDNA* genes, and high doses of N and phosphorus fertilizers decreased the abundance of diazotrophic bacteria populations, but low amounts of N and phosphorus fertilizers did not reduce their abundance (Zhou et al., 2021). The structure and abundance of the endophytic bacterial community in sweet sorghum were found to be affected by the amplification of *rrs* and *nifH* genes based on PCR-DGGE, qPCR, and high-throughput sequencing, but the diversity was not affected (Mareque et al., 2018). The diazotrophic bacterium *Paenibacillus triticisoli* BJ-18 was continuously used as an inoculum in winter wheat planted for three consecutive years, and the results showed that the abundance of some nitrogenase genes was increased significantly by using metagenome sequencing technology (Li et al., 2021a). In this study, we found that the abundance of N-fixing functional genes *nifDHK* in sugarcane inoculated with strain ED5 was significantly increased at medium N levels, whereas there was no significant difference under low and high N conditions, indicating that appropriate application of N fertilizer is beneficial to improve the associative N-fixing bacteria in sugarcane.

Nitrification oxidizes ammonia into nitrate, which can weaken the N retention capacity of the soil and accelerate the loss of N in agricultural production through nitrate leaching. This process impacts the N uptake and N use efficiency in plants and affects the N cycle in the soil environment (Subbarao et al., 2015). Nitrification is a two-step process involving the oxidation of ammonia and nitrite by bacteria to eventually produce nitrate (Van Kessel et al., 2015). Ammonia monooxygenase plays a key catalytic role in the nitrification process, and its encoding genes are *AmoABC*. It has been reported that the net nitrification rate of N in vegetable rhizosphere soil was significantly increased under single N fertilization, and ammonia-oxidizing bacteria were correlated with the net nitrification rate (Bi et al., 2017). In this study, the abundance of *AmoABC* genes was significantly decreased after inoculation with strain ED5 in the rhizosphere of sugarcane under the low N condition, whereas it increased dramatically at the high N level. Rehr and Klemme (1989) reported that applying chemical fertilizers may lead to increased denitrification and reduced ${\text{NO}}_{3}^{-}$-N to N_2_, cause N loss, and accumulate a large amount of nitrous oxide in the soil. In this study, the abundance of *NarGHI, NapAB, NiKS*, and *NorBC* genes related to the denitrification process in sugarcane rhizosphere was significantly increased after inoculation with strain ED5 under the high N condition, indicating that inoculation with strain ED5 might have induced the nitrification or denitrification process in sugarcane rhizosphere under the high N condition, which was not conducive to N fixation. The dissimilatory reduction of nitrate to ammonium (DNRA) uses two enzymes (NaR and NiR) to reduce nitrate to ammonium salts, producing cationic ammonium, which retains N in the soil due to its weak mobility. The bacteria catabolize the carbon source and have a higher utilization rate of electron acceptors (Kraft et al., 2014). In the present study, the abundance of nitrite reductase (NADH)-encoding genes *NirBD* increased significantly in the sugarcane rhizosphere in the high and medium N treatments inoculated with strain ED5, indicating that the role of strain ED5 can effectively alleviate the loss of N in the soil. When ammonium is limited in soil, some bacteria grow with nitrate or nitrite as the only N source and synthesize nitrate into ammonia with the nitrate/nitrite assimilation reductase system (NAS system), which is controlled by *NAS* gene cluster regulation (Kraft et al., 2014). In this study, the abundance of nitrate reductase catalytic subunit genes *NasAB* and nitrite ferredoxin reductase gene *NirA* were significantly increased after inoculation with strain ED5 at the high N level, indicating that strain ED5 accelerated the assimilation of nitrate under N fertilization condition, which is beneficial to improve the N use efficiency in sugarcane.

## Conclusion

In this study, the sugarcane variety GT11 was inoculated with *E. roggenkampii* ED5 at different N levels. The results showed that the IAA content in sugarcane significantly increased at the seedling stage, and the activities of N-metabolism-related enzymes showed a downward trend, but the activities of NADH and GS significantly increased under high N condition after ED5 inoculation. The total N content and N fixation efficiency in sugarcane were significantly improved after inoculating strain ED5 under low N condition. There were differences in N-metabolism-related microbial community compositions in sugarcane rhizosphere soil under low and high N application conditions after inoculation of strain ED5 at the genus level, and the N-metabolism-related microorganisms under high N condition were more different than those at medium and low N levels. The enzyme activity and abundance of N-metabolism-related genes in the sugarcane rhizosphere can be enhanced by inoculation with strain ED5, especially nitrogenase genes. It is concluded that the medium N condition was good for the biological N fixation of sugarcane with endophytic diazotroph strain ED5, which could be suggested for sugarcane production.

## Data availability statement

The datasets presented in this study can be found in online repositories. The names of the repository/repositories and accession number(s) can be found below: https://www.ncbi.nlm.nih.gov/, PRJNA911313.

## Author contributions

D-JG, D-PL, and Y-RL: planned the proposal and experiments. Y-RL and X-PS: study and resources. D-JG, D-PL, YQ, BY, PS, and RS: accomplished the experiments. AS, QK, KV, B-QZ, and X-PS: data examination. D-JG, QK, and KV: writing the original manuscript. Y-RL: review and editing. All authors contributed to the article and approved the submitted version.
