# Corrigendum: Effect of endophytic diazotroph *Enterobacter roggenkampii* ED5 on nitrogen-metabolism-related microecology in the sugarcane rhizosphere at different nitrogen levels

**DOI:** 10.3389/fmicb.2023.1290575

**Published:** 2023-09-27

**Authors:** Dao-Jun Guo, Dong-Ping Li, Bin Yang, Krishan K. Verma, Rajesh Kumar Singh, Pratiksha Singh, Qaisar Khan, Anjney Sharma, Ying Qin, Bao-Qing Zhang, Xiu-Peng Song, Yang-Rui Li

**Affiliations:** ^1^College of Life Sciences and Engineering, Hexi University, Zhangye, Gansu, China; ^2^Key Laboratory of Sugarcane Biotechnology and Genetic Improvement (Guangxi), Ministry of Agriculture, Guangxi Key Laboratory of Sugarcane Genetic Improvement, Sugarcane Research Institute, Guangxi Academy of Agricultural Sciences, Nanning, Guangxi, China; ^3^Microbiology Institute, Guangxi Academy of Agricultural Sciences, Nanning, Guangxi, China; ^4^College of Agriculture, Guangxi University, Nanning, Guangxi, China

**Keywords:** *Enterobacter roggenkampii* ED5, sugarcane growth, nitrogen fixation, rhizosphere, metagenomics, nitrogen levels

In the published article, there was an error in the author list, and author Zhuan-Di Chen was erroneously included. The corrected author list appears below.

Dao-Jun Guo^1,2†^, Dong-Ping Li^3†^, Bin Yang^1^, Krishan K. Verma^2^, Rajesh Kumar Singh^2^, Pratiksha Singh^2^, Qaisar Khan^4^, Anjney Sharma^2^, Ying Qin^4^, Bao-Qing Zhang^2^, Xiu-Peng Song^2^^*^, Yang-Rui Li^2*^

In the **Author contributions** section, “Z-DC” has been removed.

The authors apologize for this error and state that this does not change the scientific conclusions of the article in any way. The original article has been updated.

