# Supplementary material for: Effect of endophytic diazotroph Enterobacter roggenkampii ED5 on nitrogen-metabolism-related microecology in the sugarcane rhizosphere at different nitrogen levels
Source: Front Microbiol. 2023 Aug 15;14:1132016. doi: 10.3389/fmicb.2023.1132016 (PMC10464614; doi:10.3389/fmicb.2023.1132016)
Supplement: Supplementary file 1 [file Data_Sheet_1.docx]

**Table S1 Sequences of primers used for qRT-PCR amplification**

| Gene | Primer | Sequence (5→3′) |
| --- | --- | --- |
| *GS* | F | TTGGCATAGAGCAAGAGT |
|  | R | TACGGCACAGTAGTAAGG |
| *NR* | F | TGATACTGATGATGAGGAGAG |
|  | R | GGACCCACACGATAATAGT |
| EF189713 | F | F: CTCTGCCCCAAGCAAAGATG |
| (referencegene, GAPDH) | R | R: TGTTGTGCAGCTAGCATTGGA |

**Table S2 qRT-PCR reaction components**

| Component | volume |
| --- | --- |
| 2×T5 Fast qPCR Mix(SYBR Green I) | 10 μL |
| 10 μM Primer F | 0.8 μL |
| 10 μM Primer R | 0.8 μL |
| Template (cDNA) | 1 μL |
| ddH_2_O | 7.4 μL |
| Total | 20 μL |

**Table S3 Sequence statistics after quality control**

| Sample | Clean reads | Clean bases (bp) | Percent in raw reads (%) | Percent in raw bases (%) |
| --- | --- | --- | --- | --- |
| GL_2 | 59632062 | 8987589127 | 96.29544546 | 96.11522401 |
| GL_1 | 62882422 | 9477193665 | 96.600966 | 96.41731133 |
| GL_3 | 54162248 | 8161142451 | 96.03101971 | 95.82721581 |
| GLE_1 | 53662406 | 8083813063 | 86.00636592 | 85.80246632 |
| GLE_3 | 61684564 | 9297224170 | 96.40864061 | 96.23118087 |
| GLE_2 | 53636562 | 8076259762 | 95.01979334 | 94.75158435 |
| GM_3 | 52017364 | 7839918626 | 96.16224076 | 95.98223134 |
| GM_2 | 58011512 | 8742446019 | 95.54628238 | 95.3576678 |
| GM_1 | 61580288 | 9281149120 | 96.34153267 | 96.16048357 |
| GME_1 | 59928724 | 9032312884 | 96.55054438 | 96.36996961 |
| GME_2 | 56967334 | 8585671411 | 96.92018262 | 96.73544732 |
| GME_3 | 56401332 | 8500582832 | 96.58695451 | 96.40529063 |
| GH_1 | 58563248 | 8826894790 | 96.86829725 | 96.69132539 |
| GH_3 | 59311790 | 8937570967 | 96.32795784 | 96.12887909 |
| GH_2 | 52965672 | 7983137190 | 96.88259844 | 96.70477903 |
| GHE_1 | 69277094 | 10441087583 | 96.64420181 | 96.46170482 |
| GHE_3 | 59493846 | 8966023429 | 96.79043556 | 96.60137794 |
| GHE_2 | 67962308 | 10244784615 | 97.19557388 | 97.02960295 |

**Table S4 The results of sequence assembly**

| Sample | Contigs | Contigs bases (bp) | N50 (bp) | N90 (bp) | Max (bp) | Min (bp) |
| --- | --- | --- | --- | --- | --- | --- |
| GL_1 | 523084 | 270240239 | 519 | 335 | 11753 | 300 |
| GL_2 | 480134 | 249079382 | 522 | 336 | 13609 | 300 |
| GL_3 | 434089 | 213693670 | 490 | 332 | 9102 | 300 |
| GLE_1 | 439305 | 213599935 | 485 | 331 | 7417 | 300 |
| GLE_2 | 440240 | 198679996 | 444 | 324 | 4519 | 300 |
| GLE_3 | 411073 | 213197174 | 527 | 333 | 8991 | 300 |
| GM_1 | 501719 | 253898410 | 508 | 334 | 10011 | 300 |
| GM_2 | 450406 | 227774156 | 510 | 334 | 19851 | 300 |
| GM_3 | 376323 | 187932138 | 503 | 333 | 7049 | 300 |
| GME_1 | 488918 | 252677323 | 522 | 336 | 5527 | 300 |
| GME_2 | 393819 | 205252994 | 533 | 334 | 5724 | 300 |
| GME_3 | 370426 | 194144809 | 538 | 334 | 6312 | 300 |
| GH_1 | 397117 | 209888180 | 539 | 335 | 8229 | 300 |
| GH_2 | 315367 | 168592057 | 549 | 335 | 9817 | 300 |
| GH_3 | 474033 | 244237739 | 517 | 335 | 10578 | 300 |
| GHE_1 | 561456 | 299960625 | 542 | 336 | 15432 | 300 |
| GHE_2 | 518602 | 284369850 | 563 | 338 | 10448 | 300 |
| GHE_3 | 415787 | 222034668 | 546 | 336 | 41978 | 300 |

**Table S5 The results of gene prediction**

| Sample | ORFs | Total length (bp) | Average length (bp) | Max (bp) | Min (bp) |
| --- | --- | --- | --- | --- | --- |
| GL_3 | 507191 | 195212856 | 384.89 | 4785 | 102 |
| GL_2 | 572223 | 227347542 | 397.31 | 6894 | 102 |
| GL_1 | 623341 | 246461130 | 395.39 | 4839 | 102 |
| GLE_2 | 498265 | 180745881 | 362.75 | 3162 | 102 |
| GLE_3 | 489304 | 194754315 | 398.02 | 4305 | 102 |
| GLE_1 | 513304 | 198309582 | 386.34 | 3306 | 102 |
| GM_1 | 591713 | 231242286 | 390.8 | 5451 | 102 |
| GM_2 | 531535 | 207450537 | 390.29 | 9240 | 102 |
| GM_3 | 441479 | 171070773 | 387.49 | 2835 | 102 |
| GME_2 | 469904 | 186817230 | 397.56 | 4989 | 102 |
| GME_1 | 582436 | 230568453 | 395.87 | 4317 | 102 |
| GME_3 | 442089 | 176449782 | 399.13 | 3087 | 102 |
| GH_2 | 380124 | 153377073 | 403.49 | 4587 | 102 |
| GH_3 | 564769 | 222713703 | 394.34 | 4023 | 102 |
| GH_1 | 475775 | 191120991 | 401.7 | 3372 | 102 |
| GHE_1 | 677022 | 273407532 | 403.84 | 8289 | 102 |
| GHE_3 | 500823 | 201877449 | 403.09 | 5502 | 102 |
| GHE_2 | 630821 | 258647079 | 410.02 | 7587 | 102 |

**Table S6** Statistics of gene number and length removing redundancy

| Gene | Total length (bp) | Average length (bp) | Catalog gene | Catalog total length (bp) | Catalog average length (bp) |
| --- | --- | --- | --- | --- | --- |
| 20465189 | 8111362842 | 396.35 | 48624 | 23435568 | 481.98 |

**Table S7 Effect of *E. roggenkampii* ED5 on total nitrogen content in sugarcane at different nitrogen application**

| Sugarcane organs | Time  (Days) | Low nitrogen | | Mmedium nitrogen | | High nitrogen | |
| --- | --- | --- | --- | --- | --- | --- | --- |
|  |  | GL | GLE | GM | GME | GH | GHE |
| Root (N%) | 60 | 0.753±0.014^e^ | 0.881±0.009^c^ | 0.852±0.012^cd^ | 0.983±0.021^a^ | 0.844±0.015^d^ | 0.941±0.017^b^ |
|  | 120 | 0.525±0.077^b^ | 0.686±0.642^b^ | 0.529±0.358^b^ | 0.819±0.013^ab^ | 0.730±0.015^b^ | 1.093±0.015^a^ |
|  | 180 | 0.605±0.060^c^ | 0.925±0.045^a^ | 0.812±0.005^b^ | 0.814±0.005^b^ | 0.659±0.035^c^ | 0.836±0.008^b^ |
|  | 240 | 0.505±0.072^c^ | 0.703±0.017^b^ | 0.745±0.034^b^ | 0.858±0.026^a^ | 0.556±0.030^c^ | 0.718±0.029^b^ |
| Stem (N%) | 60 | 0.972±0.079^c^ | 1.301±0.045^a^ | 1.144±0.028^b^ | 1.275±0.013^a^ | 1.141±0.010^b^ | 1.276±0.014^a^ |
|  | 120 | 0.267±0.046^d^ | 0.484±0.016^c^ | 0.550±0.030^b^ | 0.774±0.013^a^ | 0.581±0.045^b^ | 0.726±0.013^a^ |
|  | 180 | 0.407±0.022^c^ | 0.541±0.035^b^ | 0.450±0.037^c^ | 0.455±0.010^c^ | 0.536±0.038^b^ | 0.652±0.027^a^ |
|  | 240 | 0.388±0.017^e^ | 0.482±0.019^c^ | 0.423±0.019^de^ | 0.475±0.040^cd^ | 0.648±0.040^b^ | 0.715±0.022^a^ |
| Leaf (N%) | 60 | 1.421±0.011^d^ | 1.467±0.014^c^ | 1.506±0.017^b^ | 1.805±0.011^a^ | 1.483±0.013^ab^ | 1.487±0.013^ab^ |
|  | 120 | 0.829±0.028^c^ | 1.089±0.072^b^ | 1.042±0.075^b^ | 1.285±0.040^a^ | 1.026±0.048^b^ | 0.997±0.023^b^ |
|  | 180 | 0.804±0.013^e^ | 0.925±0.006^d^ | 1.039±0.025^c^ | 1.033±0.024^c^ | 1.140±0.002^b^ | 1.200±0.003^a^ |
|  | 240 | 0.721±0.033^f^ | 0.797±0.006^e^ | 0.934±0.059^d^ | 1.012±0.017^c^ | 1.124±0.012^b^ | 1.185±0.024^a^ |

The data are presented as mean ±SE (n = 3, Duncan multiple range test). The means with the same alphabets in the same line are not significantly different at *p*< 0.05.

**Table S8 The results of LDA nitrogen metabolism microbial difference calculation**

| **Microorganism name** | **Group** | **LDA**  **value** | **Pvalue** |
| --- | --- | --- | --- |
| p__Proteobacteria | GH | 4.57 | 0.048 |
| p__Proteobacteria.c__Betaproteobacteria | GH | 4.04 | 0.038 |
| p__Proteobacteria.c__Betaproteobacteria.o__unclassified_c__Betaproteobacteria | GH | 3.93 | 0.030 |
| p__Proteobacteria.c__Betaproteobacteria.o__unclassified_c__Betaproteobacteria.f__unclassified_c__Betaproteobacteria | GH | 3.93 | 0.030 |
| p__Proteobacteria.c__Betaproteobacteria.o__unclassified_c__Betaproteobacteria.f__unclassified_c__Betaproteobacteria.g__unclassified_c__Betaproteobacteria | GH | 3.86 | 0.030 |
| p__Proteobacteria.c__Gammaproteobacteria | GH | 3.82 | 0.039 |
| p__Proteobacteria.c__Betaproteobacteria.o__unclassified_c__Betaproteobacteria.f__unclassified_c__Betaproteobacteria.g__unclassified_c__Betaproteobacteria.s__Betaproteobacteria_bacterium | GH | 3.67 | 0.034 |
| p__Proteobacteria.c__Deltaproteobacteria | GH | 3.66 | 0.037 |
| p__Proteobacteria.c__Deltaproteobacteria.o__unclassified_c__Deltaproteobacteria.f__unclassified_c__Deltaproteobacteria.g__unclassified_c__Deltaproteobacteria | GH | 3.44 | 0.038 |
| p__Proteobacteria.c__Deltaproteobacteria.o__unclassified_c__Deltaproteobacteria | GH | 3.40 | 0.038 |
| p__Proteobacteria.c__Deltaproteobacteria.o__unclassified_c__Deltaproteobacteria.f__unclassified_c__Deltaproteobacteria | GH | 3.32 | 0.038 |
| p__Proteobacteria.c__Deltaproteobacteria.o__unclassified_c__Deltaproteobacteria.f__unclassified_c__Deltaproteobacteria.g__unclassified_c__Deltaproteobacteria.s__Deltaproteobacteria_bacterium | GH | 3.27 | 0.034 |
| p__Proteobacteria.c__unclassified_p__Proteobacteria.o__unclassified_p__Proteobacteria.f__unclassified_p__Proteobacteria | GH | 3.12 | 0.021 |
| p__Proteobacteria.c__unclassified_p__Proteobacteria.o__unclassified_p__Proteobacteria.f__unclassified_p__Proteobacteria.g__unclassified_p__Proteobacteria | GH | 3.10 | 0.021 |
| p__Proteobacteria.c__unclassified_p__Proteobacteria | GH | 3.08 | 0.021 |
| p__Proteobacteria.c__unclassified_p__Proteobacteria.o__unclassified_p__Proteobacteria | GH | 3.07 | 0.021 |
| p__Proteobacteria.c__Betaproteobacteria.o__unclassified_c__Betaproteobacteria.f__unclassified_c__Betaproteobacteria.g__unclassified_c__Betaproteobacteria.s__Betaproteobacteria_bacterium_RIFCSPLOWO2_12_FULL_65_14 | GH | 3.05 | 0.021 |
| p__Proteobacteria.c__unclassified_p__Proteobacteria.o__unclassified_p__Proteobacteria.f__unclassified_p__Proteobacteria.g__unclassified_p__Proteobacteria.s__Proteobacteria_bacterium | GH | 3.03 | 0.023 |
| p__Proteobacteria.c__Alphaproteobacteria.o__Micropepsales.f__Micropepsaceae.g__Rhizomicrobium | GH | 2.92 | 0.023 |
| p__Proteobacteria.c__Alphaproteobacteria.o__Micropepsales | GH | 2.91 | 0.023 |
| p__Proteobacteria.c__Alphaproteobacteria.o__Micropepsales.f__Micropepsaceae | GH | 2.91 | 0.023 |
| p__Actinobacteria.c__Thermoleophilia.o__Solirubrobacterales.f__unclassified_o__Solirubrobacterales.g__unclassified_o__Solirubrobacterales.s__Solirubrobacterales_bacterium_70_9 | GH | 2.72 | 0.026 |
| p__Proteobacteria.c__Alphaproteobacteria.o__unclassified_c__Alphaproteobacteria.f__unclassified_c__Alphaproteobacteria.g__unclassified_c__Alphaproteobacteria.s__Alphaproteobacteria_bacterium_62_8 | GH | 2.69 | 0.039 |
| p__Proteobacteria.c__Deltaproteobacteria.o__Myxococcales.f__Kofleriaceae | GH | 2.67 | 0.050 |
| p__Actinobacteria.c__Actinobacteria.o__Streptomycetales.f__Streptomycetaceae.g__Streptomyces.s__unclassified_g__Streptomyces | GH | 2.67 | 0.038 |
| p__Proteobacteria.c__Alphaproteobacteria.o__Micropepsales.f__Micropepsaceae.g__Rhizomicrobium.s__Rhizomicrobium_sp__SG733 | GH | 2.50 | 0.023 |
| p__Proteobacteria.c__Betaproteobacteria.o__Burkholderiales.f__Oxalobacteraceae.g__Noviherbaspirillum.s__Noviherbaspirillum_autotrophicum | GH | 2.26 | 0.042 |
| p__Proteobacteria.c__Deltaproteobacteria.o__Myxococcales.f__Polyangiaceae.g__Polyangium.s__Polyangium_sp__SDU3_1 | GH | 2.21 | 0.037 |
| p__Candidatus_Rokubacteria.c__unclassified_p__Candidatus_Rokubacteria.o__unclassified_p__Candidatus_Rokubacteria.f__unclassified_p__Candidatus_Rokubacteria.g__unclassified_p__Candidatus_Rokubacteria.s__Candidatus_Rokubacteria_bacterium_RIFCSPHIGHO2_12_FULL_73_22 | GH | 2.20 | 0.029 |
| p__Proteobacteria.c__Gammaproteobacteria.o__Xanthomonadales.f__Rhodanobacteraceae.g__Dokdonella.s__Dokdonella_immobilis | GH | 2.12 | 0.028 |
| p__Actinobacteria.c__Actinobacteria.o__Streptomycetales.f__Streptomycetaceae.g__Streptomyces.s__Streptomyces_sp__YC419 | GH | 2.06 | 0.013 |
| p__Actinobacteria.c__Actinobacteria.o__Corynebacteriales.f__Mycobacteriaceae.g__Mycolicibacter | GH | 2.04 | 0.043 |
| p__Firmicutes.c__Clostridia.o__Clostridiales.f__Peptococcaceae.g__Desulfofundulus | GH | 2.04 | 0.048 |
| p__Proteobacteria.c__Alphaproteobacteria.o__Rhizobiales.f__Bradyrhizobiaceae.g__Nitrobacter | GH | 2.02 | 0.033 |
| p__Actinobacteria.c__Actinobacteria.o__Propionibacteriales.f__Nocardioidaceae.g__Nocardioides | GHE | 3.82 | 0.028 |
| p__Proteobacteria.c__Alphaproteobacteria.o__Sphingomonadales.f__Sphingomonadaceae | GHE | 3.26 | 0.041 |
| p__Verrucomicrobia.c__Verrucomicrobiae.o__Verrucomicrobiales | GHE | 3.07 | 0.033 |
| p__Verrucomicrobia.c__Verrucomicrobiae | GHE | 3.04 | 0.033 |
| p__Proteobacteria.c__Gammaproteobacteria.o__Xanthomonadales.f__Rhodanobacteraceae.g__Mizugakiibacter.s__Mizugakiibacter_sediminis | GHE | 2.96 | 0.017 |
| p__Proteobacteria.c__Gammaproteobacteria.o__Xanthomonadales.f__Rhodanobacteraceae.g__Mizugakiibacter | GHE | 2.93 | 0.017 |
| p__Proteobacteria.c__Gammaproteobacteria.o__Xanthomonadales.f__Rhodanobacteraceae | GHE | 2.92 | 0.031 |
| p__Proteobacteria.c__Candidatus_Muproteobacteria.o__unclassified_c__Candidatus_Muproteobacteria.f__unclassified_c__Candidatus_Muproteobacteria | GHE | 2.78 | 0.032 |
| p__Proteobacteria.c__Candidatus_Muproteobacteria.o__unclassified_c__Candidatus_Muproteobacteria.f__unclassified_c__Candidatus_Muproteobacteria.g__unclassified_c__Candidatus_Muproteobacteria | GHE | 2.77 | 0.032 |
| p__Proteobacteria.c__Candidatus_Muproteobacteria | GHE | 2.76 | 0.032 |
| p__Proteobacteria.c__Candidatus_Muproteobacteria.o__unclassified_c__Candidatus_Muproteobacteria | GHE | 2.74 | 0.032 |
| p__Proteobacteria.c__Gammaproteobacteria.o__Enterobacterales.f__Enterobacteriaceae | GHE | 2.69 | 0.031 |
| p__Verrucomicrobia.c__Verrucomicrobiae.o__Verrucomicrobiales.f__Verrucomicrobiaceae | GHE | 2.65 | 0.024 |
| p__Verrucomicrobia.c__Verrucomicrobiae.o__Verrucomicrobiales.f__unclassified_o__Verrucomicrobiales | GHE | 2.61 | 0.044 |
| p__Verrucomicrobia.c__Verrucomicrobiae.o__Verrucomicrobiales.f__unclassified_o__Verrucomicrobiales.g__unclassified_o__Verrucomicrobiales | GHE | 2.59 | 0.044 |
| p__Proteobacteria.c__Alphaproteobacteria.o__Micropepsales.f__Micropepsaceae.g__Rhizomicrobium.s__Rhizomicrobium_electricum | GHE | 2.59 | 0.031 |
| p__Proteobacteria.c__Gammaproteobacteria.o__Enterobacterales.f__Enterobacteriaceae.g__Enterobacter | GHE | 2.57 | 0.006 |
| p__Verrucomicrobia.c__Verrucomicrobiae.o__Verrucomicrobiales.f__unclassified_o__Verrucomicrobiales.g__unclassified_o__Verrucomicrobiales.s__Verrucomicrobiales_bacterium_VVV1 | GHE | 2.52 | 0.013 |
| p__Proteobacteria.c__Gammaproteobacteria.o__Enterobacterales.f__Enterobacteriaceae.g__Enterobacter.s__unclassified_g__Enterobacter | GHE | 2.41 | 0.034 |
| p__Actinobacteria.c__Actinobacteria.o__Micrococcales.f__Intrasporangiaceae.g__Phycicoccus.s__Phycicoccus_dokdonensis | GHE | 2.31 | 0.018 |
| p__Actinobacteria.c__Actinobacteria.o__Propionibacteriales.f__Nocardioidaceae.g__Nocardioides.s__Nocardioides_sp_ | GHE | 2.29 | 0.026 |
| p__Verrucomicrobia.c__Verrucomicrobiae.o__Verrucomicrobiales.f__Verrucomicrobiaceae.g__Luteolibacter | GHE | 2.27 | 0.033 |
| p__Verrucomicrobia.c__Verrucomicrobiae.o__Verrucomicrobiales.f__Verrucomicrobiaceae.g__Luteolibacter.s__Luteolibacter_sp__G_1_1_1 | GHE | 2.27 | 0.033 |
| p__Proteobacteria.c__Alphaproteobacteria.o__Rhizobiales.f__Hyphomicrobiaceae.g__Devosia.s__Devosia_sp__67_54 | GHE | 2.27 | 0.032 |
| p__Bacteroidetes.c__Cytophagia.o__Cytophagales.f__Fulvivirgaceae | GHE | 2.22 | 0.034 |
| p__Proteobacteria.c__Gammaproteobacteria.o__Enterobacterales.f__Enterobacteriaceae.g__Enterobacter.s__Enterobacter_roggenkampii | GHE | 2.18 | 0.012 |
| p__Proteobacteria.c__Betaproteobacteria.o__Nitrosomonadales.f__Nitrosomonadaceae.g__Nitrosomonas.s__Nitrosomonas_communis | GHE | 2.16 | 0.045 |
| p__Proteobacteria.c__Alphaproteobacteria.o__Rhizobiales.f__Hyphomicrobiaceae.g__Devosia.s__Devosia_sp__66_14 | GHE | 2.10 | 0.035 |
| p__Actinobacteria.c__Actinobacteria.o__Micrococcales.f__Intrasporangiaceae.g__Terrabacter.s__Terrabacter_sp__Soil810 | GHE | 2.03 | 0.028 |
| p__Proteobacteria.c__Betaproteobacteria.o__Burkholderiales.f__Comamonadaceae.g__Variovorax.s__Variovorax_sp__T529 | GHE | 2.02 | 0.048 |
| p__Actinobacteria.c__Actinobacteria.o__unclassified_c__Actinobacteria | GL | 4.12 | 0.041 |
| p__Acidobacteria.c__unclassified_p__Acidobacteria | GL | 4.12 | 0.039 |
| p__Acidobacteria.c__unclassified_p__Acidobacteria.o__unclassified_p__Acidobacteria.f__unclassified_p__Acidobacteria.g__unclassified_p__Acidobacteria | GL | 4.07 | 0.039 |
| p__Acidobacteria.c__unclassified_p__Acidobacteria.o__unclassified_p__Acidobacteria.f__unclassified_p__Acidobacteria | GL | 4.06 | 0.039 |
| p__Actinobacteria.c__Actinobacteria.o__unclassified_c__Actinobacteria.f__unclassified_c__Actinobacteria | GL | 4.05 | 0.041 |
| p__Acidobacteria.c__unclassified_p__Acidobacteria.o__unclassified_p__Acidobacteria | GL | 4.02 | 0.039 |
| p__Proteobacteria.c__Alphaproteobacteria.o__Rhizobiales | GL | 3.91 | 0.048 |
| p__Actinobacteria.c__Thermoleophilia | GL | 3.80 | 0.016 |
| p__Actinobacteria.c__Thermoleophilia.o__Solirubrobacterales | GL | 3.75 | 0.018 |
| p__Gemmatimonadetes_d__Bacteria.c__unclassified_p__Gemmatimonadetes_d__Bacteria.o__unclassified_p__Gemmatimonadetes_d__Bacteria.f__unclassified_p__Gemmatimonadetes_d__Bacteria.g__unclassified_p__Gemmatimonadetes_d__Bacteria | GL | 3.56 | 0.031 |
| p__Gemmatimonadetes_d__Bacteria | GL | 3.56 | 0.021 |
| p__Gemmatimonadetes_d__Bacteria.c__unclassified_p__Gemmatimonadetes_d__Bacteria.o__unclassified_p__Gemmatimonadetes_d__Bacteria.f__unclassified_p__Gemmatimonadetes_d__Bacteria | GL | 3.50 | 0.031 |
| p__Gemmatimonadetes_d__Bacteria.c__unclassified_p__Gemmatimonadetes_d__Bacteria | GL | 3.49 | 0.031 |
| p__Gemmatimonadetes_d__Bacteria.c__unclassified_p__Gemmatimonadetes_d__Bacteria.o__unclassified_p__Gemmatimonadetes_d__Bacteria | GL | 3.47 | 0.031 |
| p__Proteobacteria.c__Alphaproteobacteria.o__unclassified_c__Alphaproteobacteria.f__unclassified_c__Alphaproteobacteria.g__unclassified_c__Alphaproteobacteria.s__Alphaproteobacteria_bacterium | GL | 3.44 | 0.019 |
| p__Actinobacteria.c__Actinobacteria.o__Streptosporangiales.f__Thermomonosporaceae | GL | 3.04 | 0.027 |
| p__Proteobacteria.c__Alphaproteobacteria.o__unclassified_c__Alphaproteobacteria.f__unclassified_c__Alphaproteobacteria.g__unclassified_c__Alphaproteobacteria.s__Alphaproteobacteria_bacterium_13_2_20CM_2_64_7 | GL | 2.71 | 0.045 |
| p__Actinobacteria.c__unclassified_p__Actinobacteria.o__unclassified_p__Actinobacteria.f__unclassified_p__Actinobacteria.g__unclassified_p__Actinobacteria.s__Actinobacteria_bacterium_13_1_20CM_3_68_9 | GL | 2.68 | 0.042 |
| p__Proteobacteria.c__Betaproteobacteria.o__Nitrosomonadales.f__unclassified_o__Nitrosomonadales.g__unclassified_o__Nitrosomonadales.s__Nitrosomonadales_bacterium_Swamp67 | GL | 2.45 | 0.049 |
| p__Actinobacteria.c__Actinobacteria.o__Micromonosporales.f__Micromonosporaceae.g__Plantactinospora.s__Plantactinospora_sp__CNZ321 | GL | 2.24 | 0.050 |
| p__Chloroflexi.c__unclassified_p__Chloroflexi.o__unclassified_p__Chloroflexi.f__unclassified_p__Chloroflexi.g__unclassified_p__Chloroflexi.s__Chloroflexi_bacterium_GWC2_73_18 | GL | 2.21 | 0.025 |
| p__Actinobacteria.c__Actinobacteria.o__Micromonosporales.f__Micromonosporaceae.g__Asanoa | GL | 2.09 | 0.018 |
| p__Actinobacteria.c__Actinobacteria.o__Propionibacteriales.f__Nocardioidaceae.g__Kribbella.s__Kribbella_sp__NEAU_SW521 | GL | 2.04 | 0.043 |
| p__Actinobacteria.c__Thermoleophilia.o__Solirubrobacterales.f__Patulibacteraceae.g__Patulibacter.s__Patulibacter_minatonensis | GL | 2.02 | 0.032 |
| p__Actinobacteria.c__Actinobacteria.o__Micromonosporales.f__Micromonosporaceae.g__Asanoa.s__Asanoa_ferruginea | GL | 2.01 | 0.030 |
| p__Proteobacteria.c__Alphaproteobacteria.o__Rhizobiales.f__Bradyrhizobiaceae | GLE | 3.45 | 0.034 |
| p__Actinobacteria.c__Actinobacteria.o__Micrococcales.f__Intrasporangiaceae.g__Phycicoccus | GLE | 3.26 | 0.043 |
| p__Actinobacteria.c__Thermoleophilia.o__Solirubrobacterales.f__Solirubrobacteraceae.g__Solirubrobacter.s__Solirubrobacter_pauli | GLE | 3.04 | 0.043 |
| p__Proteobacteria.c__Betaproteobacteria.o__Nitrosomonadales | GLE | 2.82 | 0.049 |
| p__Actinobacteria.c__Actinobacteria.o__Propionibacteriales.f__Nocardioidaceae.g__Marmoricola.s__Marmoricola_caldifontis | GLE | 2.67 | 0.023 |
| p__Proteobacteria.c__Betaproteobacteria.o__Nitrosomonadales.f__unclassified_o__Nitrosomonadales.g__unclassified_o__Nitrosomonadales | GLE | 2.57 | 0.021 |
| p__Actinobacteria.c__Actinobacteria.o__Micrococcales.f__Intrasporangiaceae.g__Knoellia.s__Knoellia_sp__DB2414S | GLE | 2.57 | 0.040 |
| p__Proteobacteria.c__Betaproteobacteria.o__Nitrosomonadales.f__unclassified_o__Nitrosomonadales | GLE | 2.55 | 0.021 |
| p__Chloroflexi.c__Chloroflexia.o__Chloroflexales.f__Roseiflexaceae.g__Roseiflexus.s__Roseiflexus_sp__RS_1 | GLE | 2.43 | 0.026 |
| p__Actinobacteria.c__Actinobacteria.o__Micrococcales.f__Intrasporangiaceae.g__Knoellia.s__Knoellia_flava | GLE | 2.34 | 0.037 |
| p__Actinobacteria.c__Actinobacteria.o__Micrococcales.f__Micrococcaceae.g__Arthrobacter.s__Arthrobacter_sp__NEB_688 | GLE | 2.08 | 0.035 |
| p__Actinobacteria.c__Thermoleophilia.o__Solirubrobacterales.f__Solirubrobacteraceae.g__Solirubrobacter | GM | 3.60 | 0.030 |
| p__Actinobacteria.c__Thermoleophilia.o__Solirubrobacterales.f__Solirubrobacteraceae | GM | 3.54 | 0.030 |
| p__Proteobacteria.c__Gammaproteobacteria.o__Nevskiales | GM | 3.49 | 0.035 |
| p__Actinobacteria.c__Actinobacteria.o__Micromonosporales | GM | 3.30 | 0.018 |
| p__Actinobacteria.c__Actinobacteria.o__Micromonosporales.f__Micromonosporaceae | GM | 3.25 | 0.018 |
| p__Actinobacteria.c__Thermoleophilia.o__Solirubrobacterales.f__Solirubrobacteraceae.g__Solirubrobacter.s__Solirubrobacter_sp__URHD0082 | GM | 2.87 | 0.046 |
| p__Proteobacteria.c__Gammaproteobacteria.o__Nevskiales.f__Steroidobacteraceae.g__Steroidobacter.s__Steroidobacter_agariperforans | GM | 2.85 | 0.031 |
| p__Proteobacteria.c__Alphaproteobacteria.o__Rhizobiales.f__Rhizobiaceae.g__Sinorhizobium.s__Sinorhizobium_meliloti | GM | 2.64 | 0.022 |
| p__Planctomycetes.c__unclassified_p__Planctomycetes.o__unclassified_p__Planctomycetes.f__unclassified_p__Planctomycetes.g__unclassified_p__Planctomycetes.s__Planctomycetes_bacterium_ETA_A1 | GM | 2.40 | 0.024 |
| p__Proteobacteria.c__Gammaproteobacteria.o__Nevskiales.f__Steroidobacteraceae.g__Steroidobacter.s__Steroidobacter_denitrificans | GM | 2.38 | 0.036 |
| p__Proteobacteria.c__Gammaproteobacteria.o__Methylococcales.f__Methylococcaceae.g__Methylobacter.s__Methylobacter_sp_ | GM | 2.34 | 0.005 |
| p__Proteobacteria.c__Gammaproteobacteria.o__Xanthomonadales.f__Xanthomonadaceae.g__Stenotrophomonas.s__unclassified_g__Stenotrophomonas | GM | 2.27 | 0.013 |
| p__Actinobacteria.c__Actinobacteria.o__Streptosporangiales.f__Streptosporangiaceae.g__Nonomuraea.s__Nonomuraea_sp__ATCC_55076 | GM | 2.25 | 0.013 |
| p__Proteobacteria.c__Gammaproteobacteria.o__Xanthomonadales.f__Xanthomonadaceae.g__Thermomonas.s__Thermomonas_hydrothermalis | GM | 2.21 | 0.005 |
| p__Proteobacteria.c__Alphaproteobacteria.o__Rhodospirillales.f__Rhodospirillaceae.g__Inquilinus | GM | 2.21 | 0.021 |
| p__Proteobacteria.c__Gammaproteobacteria.o__Pseudomonadales.f__Pseudomonadaceae.g__Pseudomonas.s__Pseudomonas_panipatensis | GM | 2.14 | 0.005 |
| p__Proteobacteria.c__Gammaproteobacteria.o__Methylococcales.f__Methylococcaceae.g__Methylobacter | GM | 2.13 | 0.048 |
| p__Proteobacteria.c__Alphaproteobacteria.o__Rhizobiales.f__Brucellaceae.g__Brucella | GM | 2.12 | 0.037 |
| p__Proteobacteria.c__Alphaproteobacteria.o__Rhizobiales.f__Brucellaceae.g__Brucella.s__Brucella_abortus | GM | 2.10 | 0.037 |
| p__Proteobacteria.c__Betaproteobacteria.o__Burkholderiales.f__Burkholderiaceae.g__Polynucleobacter.s__Polynucleobacter_wuianus | GM | 2.05 | 0.020 |
| p__Proteobacteria.c__Betaproteobacteria.o__Burkholderiales | GME | 3.42 | 0.050 |
| p__Proteobacteria.c__Betaproteobacteria.o__Burkholderiales.f__Comamonadaceae.g__Ramlibacter | GME | 2.73 | 0.031 |
| p__Proteobacteria.c__Oligoflexia | GME | 2.70 | 0.017 |
| p__Proteobacteria.c__Oligoflexia.o__Bdellovibrionales | GME | 2.70 | 0.017 |
| p__Proteobacteria.c__Oligoflexia.o__Bdellovibrionales.f__Bdellovibrionaceae | GME | 2.68 | 0.013 |
| p__Proteobacteria.c__Oligoflexia.o__Bdellovibrionales.f__Bdellovibrionaceae.g__Bdellovibrio | GME | 2.67 | 0.013 |
| p__Actinobacteria.c__Actinobacteria.o__Micromonosporales.f__Micromonosporaceae.g__Actinoplanes | GME | 2.67 | 0.039 |
| p__Proteobacteria.c__Oligoflexia.o__Bdellovibrionales.f__Bdellovibrionaceae.g__Bdellovibrio.s__Bdellovibrio_bacteriovorus | GME | 2.36 | 0.016 |
| p__Proteobacteria.c__Gammaproteobacteria.o__Oceanospirillales.f__Oceanospirillaceae | GME | 2.28 | 0.019 |
| p__Proteobacteria.c__Gammaproteobacteria.o__Pseudomonadales.f__Pseudomonadaceae.g__Pseudomonas.s__Pseudomonas_sp__2FE | GME | 2.28 | 0.016 |
| p__Firmicutes.c__unclassified_p__Firmicutes.o__unclassified_p__Firmicutes.f__unclassified_p__Firmicutes.g__unclassified_p__Firmicutes.s__Firmicutes_bacterium_HGW_Firmicutes_12 | GME | 2.14 | 0.047 |
| p__Proteobacteria.c__Oligoflexia.o__Bdellovibrionales.f__Bdellovibrionaceae.g__Bdellovibrio.s__Bdellovibrio_sp__SKB1291214 | GME | 2.09 | 0.021 |
| p__Verrucomicrobia.c__Verrucomicrobiae.o__Verrucomicrobiales.f__Akkermansiaceae.g__unclassified_f__Akkermansiaceae | GME | 2.04 | 0.035 |
| p__Proteobacteria.c__Oligoflexia.o__Bdellovibrionales.f__Bdellovibrionaceae.g__Bdellovibrio.s__Bdellovibrio_sp__ArHS | GME | 2.04 | 0.024 |
| p__Verrucomicrobia.c__Verrucomicrobiae.o__Verrucomicrobiales.f__Akkermansiaceae.g__unclassified_f__Akkermansiaceae.s__Akkermansiaceae_bacterium | GME | 2.04 | 0.035 |
